# Prox1 Is Required for Granule Cell Maturation and Intermediate Progenitor Maintenance During Brain Neurogenesis

**DOI:** 10.1371/journal.pbio.1000460

**Published:** 2010-08-17

**Authors:** Alfonso Lavado, Oleg V. Lagutin, Lionel M. L. Chow, Suzanne J. Baker, Guillermo Oliver

**Affiliations:** 1 Department of Genetics & Tumor Cell Biology, St. Jude Children's Research Hospital, Memphis, Tennessee, United States of America; 2 Department of Developmental Neurobiology, St. Jude Children's Research Hospital, Memphis, Tennessee, United States of America; Stanford University, United States of America

## Abstract

The dentate gyrus has an important role in learning and memory, and adult neurogenesis in the subgranular zone of the dentate gyrus may play a role in the acquisition of new memories. The homeobox gene *Prox1* is expressed in the dentate gyrus during embryonic development and adult neurogenesis. Here we show that Prox1 is necessary for the maturation of granule cells in the dentate gyrus during development and for the maintenance of intermediate progenitors during adult neurogenesis. We also demonstrate that Prox1-expressing intermediate progenitors are required for adult neural stem cell self-maintenance in the subgranular zone; thus, we have identified a previously unknown non-cell autonomous regulatory feedback mechanism that controls adult neurogenesis in this region of the mammalian brain. Finally, we show that the ectopic expression of Prox1 induces premature differentiation of neural stem cells.

## Abstract 2

The transcription factor Prox1 plays a crucial role in intermediate progenitor maintenance and hippocampal neuron differentiation during adult neurogenesis in the dentate gyrus region of the hippocampus.

## Author Summary

In the brain, the hippocampus has a crucial role in learning and memory. In mammals, neurogenesis (the birth of new neurons) occurs in the dentate gyrus region of the hippocampus throughout adulthood, and this activity is thought to be the basis for the acquisition of new memories. In this study we describe for the first time the functional roles of the transcription factor *Prox1* during brain development and adult neurogenesis. We demonstrate that in mammals, *Prox1* is required for the differentiation of granule cells during dentate gyrus development. We also show that conditional inactivation of *Prox1* results in the absence of specific intermediate progenitors in the subgranular zone of the dentate gyrus, which prevents adult neurogenesis from occurring. This is the first report showing blockade of adult neurogenesis at the level of progenitor cells. Next, we demonstrate that in the absence of Prox1-expressing intermediate progenitors, the stem cell population of the subgranular zone becomes depleted. Further, we show that Prox1-expressing intermediate progenitors are required for adult neural stem cell self-maintenance in the subgranular zone. Finally, we demonstrate that Prox1 ectopic expression induces premature granule cell differentiation in the subgranular zone. Therefore, our results identify a previously unknown non-cell autonomous feedback mechanism that links adult stem cell self-maintenance with neuronal differentiation in the dentate gyrus and could have important implications for neurogenesis in other brain regions.

## Introduction

In the brain, the dentate gyrus (DG) is the primary afferent pathway into the hippocampus. The DG has a crucial role in learning and memory [1],[2],[3]. In mammals, neurogenesis occurs in the subgranular zone (SGZ) of the DG throughout adulthood [4],[5],[6],[7]; this activity is thought to be the basis for the acquisition of new memories [3],[8],[9].

The formation of the DG is a complex process that involves cell migration and neuronal differentiation [10],[11]. Factors that regulate DG development are thought to have a similar function during adult neurogenesis. In the SGZ, astrocyte-like adult neural stem cells (NSCs) give rise to a series of intermediate progenitors that eventually differentiate into neurons [12]. Several signaling molecules, including Wnt, Noggin/BMP, Shh, and Notch, regulate adult NSC self-maintenance, proliferation, and progenitor differentiation [13],[14]. However, little is known about how the generation of the proper number of descendants is controlled. It has been proposed that once generated, NSC descendants can trigger some type of feedback mechanism to stop stem cell differentiation [15]. In this context, Notch signaling has been considered a candidate to regulate such a feedback mechanism during adult neurogenesis [13].

The homeobox gene *Prox1* is expressed in several brain regions (i.e., cortex, DG, thalamus, hypothalamus, cerebellum) during prenatal and postnatal stages of development [16],[17],[18]. Interestingly, *Prox1* is expressed throughout all stages of DG development and in adult granule cells; therefore, Prox1 is commonly used as a specific marker for these cells [15],[19]. However, no data are yet available on the functional role(s) of Prox1 during brain development.

We have now determined that functional inactivation of *Prox1* during DG development results in defective granule cell maturation and the loss of this cell population. We also report that conditional inactivation of *Prox1* in the SGZ during adult neurogenesis leads to the lack of intermediate progenitors, and as a consequence, the disruption of the mechanism involved in NSC self-maintenance. Therefore, we have identified a previously unknown non-cell autonomous regulatory feedback mechanism that links adult NSC self-maintenance with the generation of the proper number of descendants in the SGZ. Finally, we show that ectopic expression of Prox1 in NSCs promotes premature differentiation during DG development and adult neurogenesis in the SGZ.

## Results

### Prox1 Activity Is Required for DG Formation

**Figure 1:**
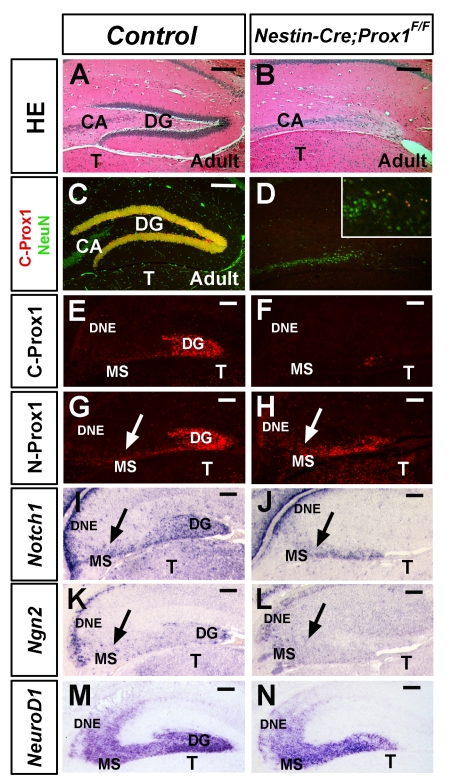
Lack of Prox1 affects dentate gyrus formation. Hematoxylin and eosin (HE) staining of coronal sections of adult hippocampus of (A) control and (B) *Nestin-Cre;Prox1^F/F^* adult brains shows the absence of a dentate gyrus (DG) in the mutant mice. (C) Mature granule cells are C-Prox1^+^ and NeuN^+^. (D) In the *Nestin-Cre;Prox1^F/F^* brain, the few remaining Prox1^+^ cells present in the hippocampal region are C-Prox1^+^ NeuN^+^ mature granule cells that escaped deletion (insert in D). (E, F) Only a few wild-type C-Prox1^+^ granule cells remain in the *Nestin-Cre;Prox1^F/F^* brain at E16.5. (G, H) There is a reduction in the number of N-Prox1^+^ cells in the *Nestin-Cre;Prox1^F/F^* brain at this stage, although these cells are still able to migrate outside the dentate neuroepithelium (DNE) (arrows). Although reduced, *Notch1* (I, J) and *Ngn2* (K, L) expression remains in the DNE and DG of *Nestin-Cre;Prox1^F/F^* embryos as shown by ISH. Moreover, *Notch1* and *Ngn2* progenitors (arrows) migrate outside of the mutant DNE. (M, N) The *NeuroD1* expression domain, although reduced, remains in the migratory stream (MS) and the DG of *Nestin-Cre;Prox1^F/F^* embryos as shown by ISH. Scale bar: 100 µm. CA, Pyramidal cell layer; T, Thalamus. Scale bar: 100 µm.

Standard *Prox1*-null embryos die during midgestation [20]; therefore, to evaluate the possible functional roles of Prox1 in the mammalian brain, we used a conditional-inactivation approach. An available *Prox1*-floxed strain [21] was initially bred with *Nestin-Cre* mice in which constitutively active Cre recombinase is expressed in neural progenitors from embryonic day (E) 10.5 [22]. Adult *Nestin-Cre;Prox1^F/F^* mice were viable but had only a few scattered Prox1^+^/NeuN^+^ wild-type granule cells in their hippocampi (Figure 1B,D). During embryonic development, *Prox1* expression is detected in both the dentate neuroepithelium (DNE) and the DG [17],[23]. Therefore, we performed a detailed characterization of the development of the DG in *Nestin-Cre;Prox1^F/F^* conditional-mutant embryos.

**Figure 2:**
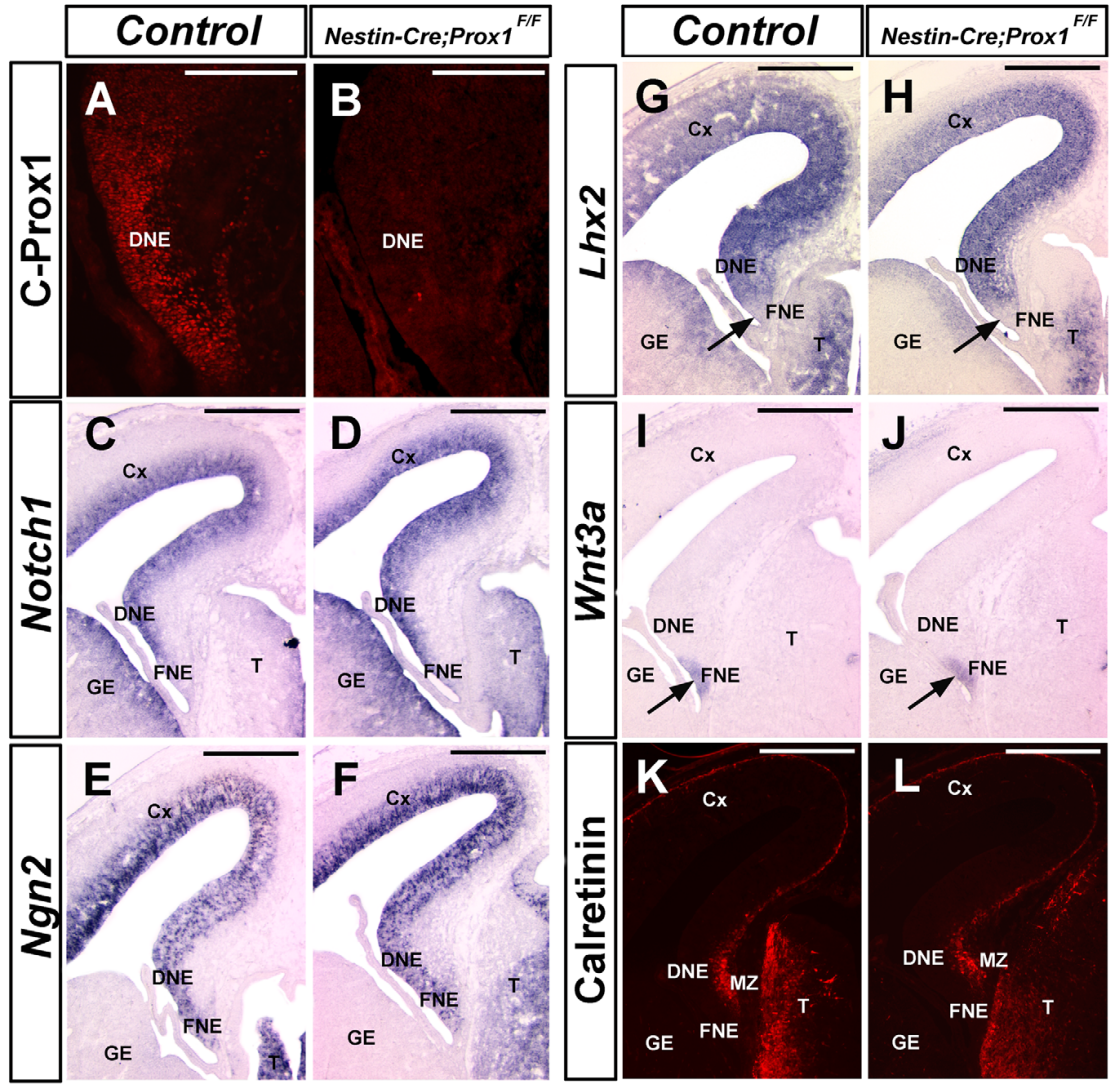
Patterning of the Ammon's horn is normal in the dentate gyrus of E14.5 *Nestin-Cre;Prox1^F/F^* embryos. (A) Prox1 is expressed in the dentate gyrus neuroepithelium (DNE) at E14.5. (B) *Nestin-Cre* successfully removed most Prox1 expression from the DNE at this stage. However, the expression pattern of *Notch1* (C, D) and *Ngn2* (E, F) is not changed in the DNE of *Nestin-Cre;Prox1^F/F^* littermates. (G, H) *Lhx2* is expressed in the developing dentate gyrus (DG) but not in the fimbria neuroepithelium (FNE, arrows) of control and *Nestin-Cre;Prox1^F/F^* brains. (I, J) *Wnt3a* expression is detected in the DG and FNE of control and conditional mutant brains at this stage (arrows). (K, L) Calretinin expression in the marginal zone (MZ) is also normal in the *Nestin-Cre;Prox1^F/F^* brain. Cx, cortex; T, thalamus; GE, ganglionic eminence. Scale bar in (A, B): 50 µm. Scale bar in (C–L): 100 µm.

At E14.5, the DG of *Nestin-Cre;Prox1^F/F^* embryos showed normal Ammon's horn formation (Figure 2A–L). At E16.5, and as shown by an anti-C-Prox1 antibody that recognizes only the wild-type form of Prox1 (see Figure S1A,B for more details), only a few cells escaped Cre-mediated deletion in the DG of *Nestin-Cre;Prox1^F/F^* embryos (Figure 1F). However, as indicated by an anti-N-Prox1 antibody that recognizes both wild-type and conditional-mutant forms of Prox1 (Figure S1A,B), the number of N-Prox1^+^ cells was reduced in the DG of *Nestin-Cre;Prox1^F/F^* embryos at this stage (Figure 1H; Figure S1C,D). Reduced numbers of *Notch1* ^+^, *Ngn2* ^+^, and *NeuroD1^+^* cells were also observed by ISH in the mutant DG (Figure 1J,L,N); however, we found no obvious alterations in the expression of *Wnt3a* in the FNE (Figure S2A,B) [24], or *lef1* in the migratory stream (MS) (Figure S2C,D) [25]. We also did not find obvious anomalies in radial glia scaffolding [26] in the mutant DG at E16.5 (Figure S2E–H). These results indicate that in the *Nestin-Cre;Prox1^F/F^* embryos, the N-Prox1^+^ cells are capable of migrating out of the DNE and reach the DG.

### Prox1 Controls Cell Proliferation in the DNE

**Figure 3:**
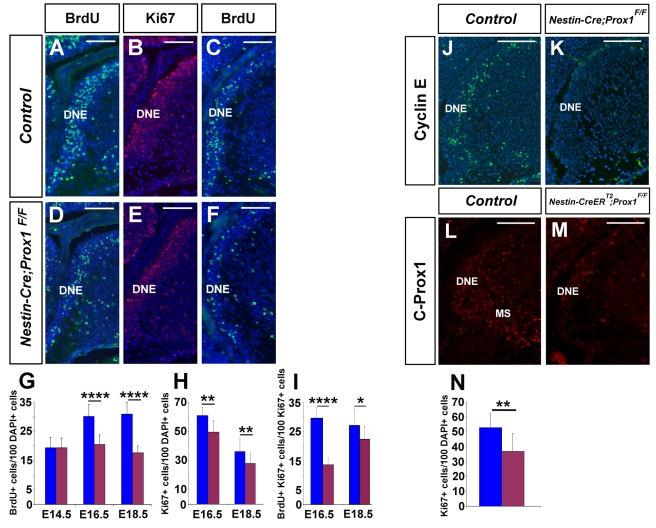
Reduced proliferation in the dentate gyrus neuroepithelium of *Nestin-Cre;Prox1^F/F^* embryos. (A, D) BrdU (after 1-h pulse) and Ki67 (B, E) immunostaining revealed reduced proliferation in the dentate gyrus neuroepithelium (DNE) of *Nestin-Cre;Prox1^F/F^* brains starting at E16.5. Similar results were obtained at E18.5 (C, F, G, H). (I) Cells in the DNE of *Nestin-Cre;Prox1^F/F^* embryos have a longer cell cycle as shown by double BrdU/Ki67 immunostaining. (J, K) Cyclin E^+^ cells are absent from the DNE of E16.5 *Nestin-Cre;Prox1^F/F^* brains. (L) C-Prox1^+^ cells are normally found in the DNE and migratory stream (MS) of control brains at E18.5. Following TM administration at E16.5, there are fewer C-Prox1^+^ cells in the DNE and MS (M) of *Nestin-CreER^T2^;Prox1^F/F^* brains. (N) As indicated by Ki67 counting, the number of cycling cells is reduced in the DNE of *Nestin-CreER^T2^;Prox1^F/F^* embryos. Data represent the mean number of positive cells per DG section ± SD (*N* = 3 embryos). Paired *t* test. * *p*<0.1; ** *p*<0.01; **** *p*<0.0001. Scale bar in (A–M): 50 µm.

Prox1 absence leads to cell cycle alterations in the developing neuroretina [27]. To evaluate possible alterations in cell proliferation during DG development, we compared the number of cycling cells in the DNE of wild-type and *Nestin-Cre;Prox1^F/F^* embryos by using a 1-h BrdU pulse starting at E14.5. Significantly fewer BrdU^+^ cells were observed in the *Nestin-Cre;Prox1^F/F^* DNE at E16.5 (Figure 3D,G) and E18.5 (Figure 3F,G). Similar results were obtained with Ki67 (Figure 3E,H). Results using BrdU/Ki67 double immunostaining (Figure 3I) determined that cells in the DNE were cycling more slowly in *Nestin-Cre;Prox1^F/F^* embryos at E16.5 and E18.5. We also found that CyclinE expression was reduced in the *Nestin-Cre;Prox1^F/F^* DNE at E16.5 (Figure 3K). To confirm that the reduced proliferation of cells in the DNE was caused by the lack of Prox1 in that region at around E16.5, we used a tamoxifen (TM)-inducible *Nestin-CreER^T2^* strain [28] to induce *Prox1* deletion later during development. Following TM administration at E16.5 (see Figure S3A for details), most C-Prox1^+^ cells were missing in the DNE and MS of E18.5 *Nestin-CreER^T2^*;*Prox1^F/F^* embryos (Figure 3M). Fewer Ki67^+^ cells were also observed in the DNE of these mutant embryos (Figure 3N). Thus, *Prox1* is required to regulate cell proliferation in the DNE during early stages of DG development.

### Granule Cell Maturation Is Arrested in the Absence of Prox1

**Figure 4:**
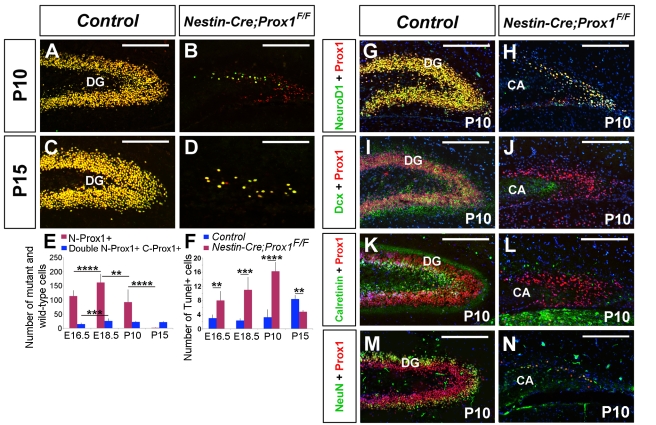
Granule cell maturation is affected in Δ-Prox1 cells. (A–E) As shown by double-staining with anti-N-Prox1 (red) and anti-C-Prox1 (green), the number of Δ-Prox1 cells (red bars in E) is reduced in the *Nestin-Cre;Prox1^F/F^* dentate gyrus (DG) at postnatal stages of development (blue bars in E represent double N-Prox1/C-Prox1^+^ wild-type cells). (F) There is an increase in the number of TUNEL^+^ cells in the mutant DG as granule cell maturation progresses. IHC using anti-N-Prox1 and anti-NeuroD1 antibodies revealed that all granule cells are NeuroD1^+^ both in P10 wild-type (G) and conditional mutant (H) DG. However, in the conditional mutant hippocampal region most of the N-Prox1^+^ cells are Dcx^−^ (J) and Calretinin^−^ (L). The only NeuN^+^ cells left in the *Nestin-Cre;Prox1^F/F^* hippocampal region are the C-Prox1^+^ cells that have escaped Cre deletion (N). Panels I, K, and M correspond to controls. Data represent the mean number of positive cells per DG section ± SD. *N* = 3 brains. Paired *t* test. ** *p*<0.01; *** *p*<0.001; **** *p*<0.0001. CA, Pyramidal cell layer. Scale bar: 100 µm.

During DG formation, *Prox1* expression is upregulated in intermediate progenitors and immature granule cells, and throughout adulthood, its expression is maintained in mature granule cells [17],[23]. Therefore, we analyzed whether Prox1 is necessary for the acquisition of granule cell identity by monitoring the Δ-Prox1 cells. Using this approach, we observed an increase in the number of Δ-Prox1 cells (cells recognized by the anti-N-Prox1 antibody but not by the anti-C-Prox1 antibody; see Figure S1A,B for details) in the DG region of *Nestin-Cre;Prox1^F/F^* embryos until E18.5 (Figure S1D,F and Figure 4E); however, at later stages, the number of Δ-Prox1 cells in the DG decreased. This reduction was particularly apparent at postnatal stages; by postnatal day (P) 15, most Prox1^+^ cells in the mutant DG corresponded to those that escaped deletion (Figure 4B,D,E). As revealed by TUNEL assay, starting at E16.5 the number of TUNEL^+^ cells increased in the DG of *Nestin-Cre;Prox1^F/F^* embryos (Figure 4F), a finding suggesting that Prox1 is necessary for the survival of intermediate progenitors and immature granule cells.

In the DG, terminal differentiation of granule cells occurs during early postnatal stages. Therefore, we analyzed whether the observed increase in the number of TUNEL^+^ cells was due to defective differentiation of Δ-Prox1 granule cells. Previous studies have shown that the bHLH protein NeuroD1 is required for the maturation of granule cells [15],[29],[30]. We found that at P10, N-Prox1^+^ cells in the mutant DG were also NeuroD1^+^ (Figure 4H); however, they did not co-express Dcx^+^ [31] or Calretinin^+^ [32] (Figure 4J,L), and only the few C-Prox1^+^ granule cells that escaped Cre deletion co-expressed NeuN (Figure 4N) [33]. These results suggest that lack of Prox1 activity arrested granule cell differentiation.

Next, we used an in vitro assay to determine whether the functional inactivation of *Prox1* affects neuronal differentiation. *Nestin-Cre;Prox1^F/+^* and *Nestin-Cre;Prox1^F/F^* neurospheres were isolated from E16.5 hippocampal regions. *Nestin-Cre;Prox1^F/F^* neurospheres produced cells that were positive for the early neuronal marker β-tubulin-III at a relatively similar rate (119 of 135) compared to that of wild-type neurospheres (159 of 175) (Figure S4A–C). However, fewer *Nestin-Cre;Prox1^F/F^* neurospheres produced Dcx^+^ cells (8 of 190), as compared with wild-type controls (199 of 216) (Figure S4D–F).

To determine whether Prox1 is necessary for *Dcx* expression, we cotransfected *Nestin-Cre;Prox1^F/F^* neurospheres with GFP-expressing and full-length *Prox1* cDNA-expressing plasmids. We found that *Nestin-Cre;Prox1^F/F^* neurospheres transfected with *Prox1* cDNA produced Dcx^+^ cells (20 of 76 GFP^+^ neurospheres), but those transfected only with the GFP-expressing plasmid did not (0 of 73 GFP^+^ neurospheres) (Figure S4G,H). Thus, as seen in vivo, Prox1 is not necessary to induce neuronal differentiation in vitro but is required for the expression of later neuronal markers such as *Dcx*.

### Prox1 Is Required for Granule Cell Maturation at Postnatal Stages

**Figure 5:**
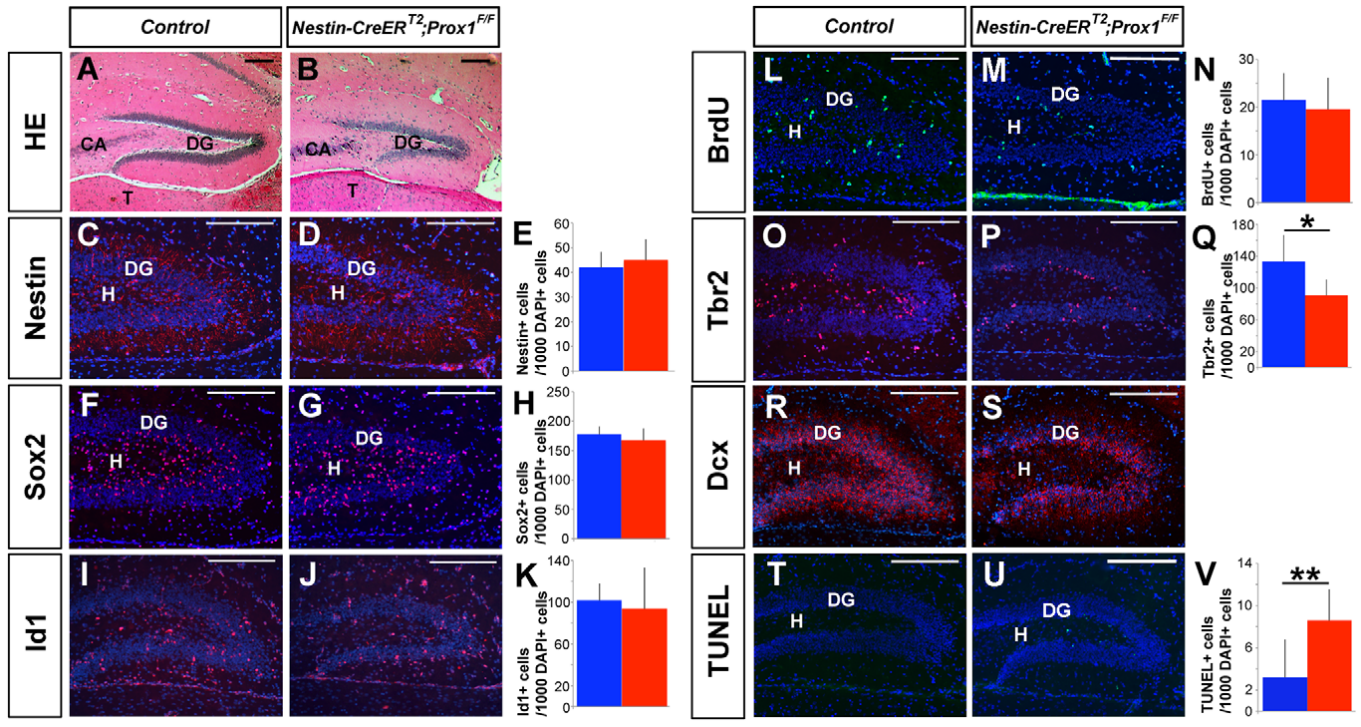
Post-natal lack of Prox1 affects dentate gyrus formation. Hematoxylin and eosin staining (A, B) of coronal sections of the hippocampus shows a reduced dentate gyrus (DG) in 2-mo-old *Nestin-CreER^T2^;Prox1^F/F^* mice treated with TM from P0 to P15 (B). There are no changes in the neural stem cell population at P10, as indicated by Nestin (C–E), Sox2 (F–H), and Id1 (I–K) IHC in the *Nestin-CreER^T2^;Prox1^F/F^* DG. BrdU staining after a 1-h pulse shows a similar level of proliferation in the DG of wild-type (L, N) and *Nestin CreER^T2^;Prox1^F/F^* brains (M, N). The number of Tbr2^+^ intermediate progenitors (O–Q) and Dcx^+^ (R, S) cells is reduced in the *Nestin-CreER^T2^;Prox1^F/F^* DG at P10. We also observed an increase in the number of TUNEL^+^ cells (T–V) in the DG area of the *Nestin-CreER^T2^;Prox1^F/F^* brains at P10. Data represent the mean number of positive cells per DG section ± SD (*N* = 3 mice). Blue bars are controls. Red bars are TM-treated *Nestin CreER^T2^;Prox1^F/F^* brains. Paired *t* test. * *p*<0.1; ** *p*<0.01. CA, pyramidal cell layer; T, thalamus; H, Hilus. Scale bar: 100 µm.

To confirm that the conditional inactivation of *Prox1* at postnatal stages is directly responsible for the defective granule cell maturation phenotype observed in *Nestin-Cre;Prox1^F/F^* mice, we next deleted *Prox1* during postnatal stages by using the *Nestin-CreER^T2^* transgenic mouse strain [28]. TM was administered to *Nestin-CreER^T2^*;*Prox1^F/F^* pups at early postnatal stages (see Figure S3B for details); at these stages, there are no Prox1^+^ NSCs in the hilus (Figure S5A–E). At 2 mo of age, TM-treated *Nestin-CreER^T2^*;*Prox1^F/F^* mice had smaller DGs than their control littermates (Figure 5B). The number of Tbr2^+^ intermediate progenitors [34] was reduced in both P5 (Figure S5R,S) and P10 (Figure 5P,Q) pups. They also exhibited fewer Dcx^+^ cells at P5 (Figure S5T,U) and P10 (Figure 5R,S). Moreover, the ratio of Tbr2^+^:Calretinin^+^ cells was increased in the TM-treated *Nestin-CreER^T2^*;*Prox1^F/F^* (1.55±0.25:1; *N* = 3) pups at P10 (control: 1.09±0.28:1; *N* = 3; *p*<0.1). These results suggest that the reduced size of the DG was caused by a reduction in the number of intermediate progenitors and by an arrest in granule cell differentiation. Moreover, as indicated by TUNEL (Figure S5V–Y and Figure 4T–V) and active Caspase-3 assays (Figure S6A,B), an increase in cell death was observed in the mutant DG at these stages. On the other hand, cell proliferation was reduced at P5 but not at P10 in the DG of *Nestin-CreER^T2^*;*Prox1^F/F^* pups (Figure S5P; Figure 5N). The number of Nestin^+^, Sox2^+^, or Id1^+^ NSCs was normal at P5 and P10 (Figure S5J,M; Figure 5C–K). These data suggest that the lack of Prox1 during postnatal stages is directly responsible for the reduction in the number of intermediate progenitors and the defect in granule cell differentiation that secondarily promotes an increase in cell death and ultimately a reduction in the size of the DG.

### Prox1 Is Required for the Maintenance of Intermediate Progenitors in the SGZ During Adult Neurogenesis

**Figure 6:**
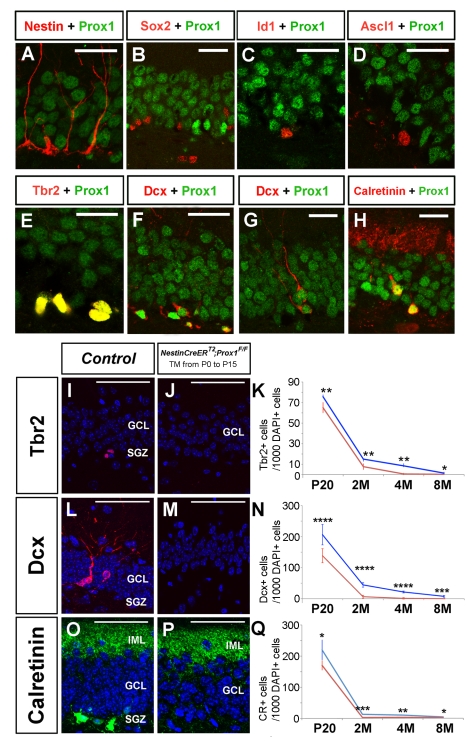
Prox1 is required in the subgranular zone during adult neurogenesis. During adult neurogenesis, Prox1 is not expressed in Nestin^+^ (A), Sox2^+^ (B), or Id1^+^ (C) adult neural stem cells or in Acsl^+^ Type-IIa intermediate progenitors (D). Instead, Prox1 is expressed in Tbr2^+^ (E) and Dcx^+^ Type-IIb intermediate progenitors (F), Type-III intermediate progenitors (G), and Calretinin^+^ immature neurons. Graphs compare the number of Tbr2^+^ (K) Dcx^+^ (N) and Calretinin^+^ (Q) cells in P20, 2-mo-old, 4-mo-old, and 8-mo-old control (blue) and *Nestin-CreER^T2^;Prox1^F/F^* (red) mice. A reduced number of Tbr2^+^ (J, K), Dcx^+^ (M, N), and Calretinin^+^ (P, Q) cells was observed in the SGZ of 4-mo-old *Nestin-CreER^T2^;Prox1^F/F^* mice. Data represent the mean number of positive cells per DG section ± SD. *N* = 3 brains. Paired *t* test. * *p*<0.1; ** *p*<0.01; *** *p*<0.001; **** *p*<0.0001. GCL, granule cell layer; IML, inner molecular layer; M, month. Scale bar in (A–H): 25 µm. Scale bar in (I, J, L, M, O, P): 50 µm.

In the SGZ, Prox1 expression is initially detected in Tbr2^+^ (Figure 6E) [35] and Dcx^+^ (Figure 6F) Type-IIb intermediate progenitors [36] and is absent from adult NSCs (Figure 6A,B,C) [37],[38] and Ascl1^+^ intermediate progenitors (Figure 6D) [36]. As neurogenesis progresses, Prox1 is detected in Calretinin^+^ cells (Figure 6H). Analysis of the SGZ of *Nestin-CreER^T2^*;*Prox1^F/F^* pups treated with TM daily from P0 to P15 (see Figure S3B for the details of the TM treatment) identified a reduced number of Tbr2^+^ (Figure 6K) and Dcx^+^ (Figure 6N) cells at P20; these cells were nearly absent at 4 and 8 mo of age (Figure 6J,K,M,N). As indicated by C-Prox1 immunostaining, the few remaining Dcx^+^ cells were those that escaped deletion (Figure S7). Fewer Calretinin^+^ immature neuron cells were also observed in the mutant brains (Figure 6P,Q).

**Figure 7:**
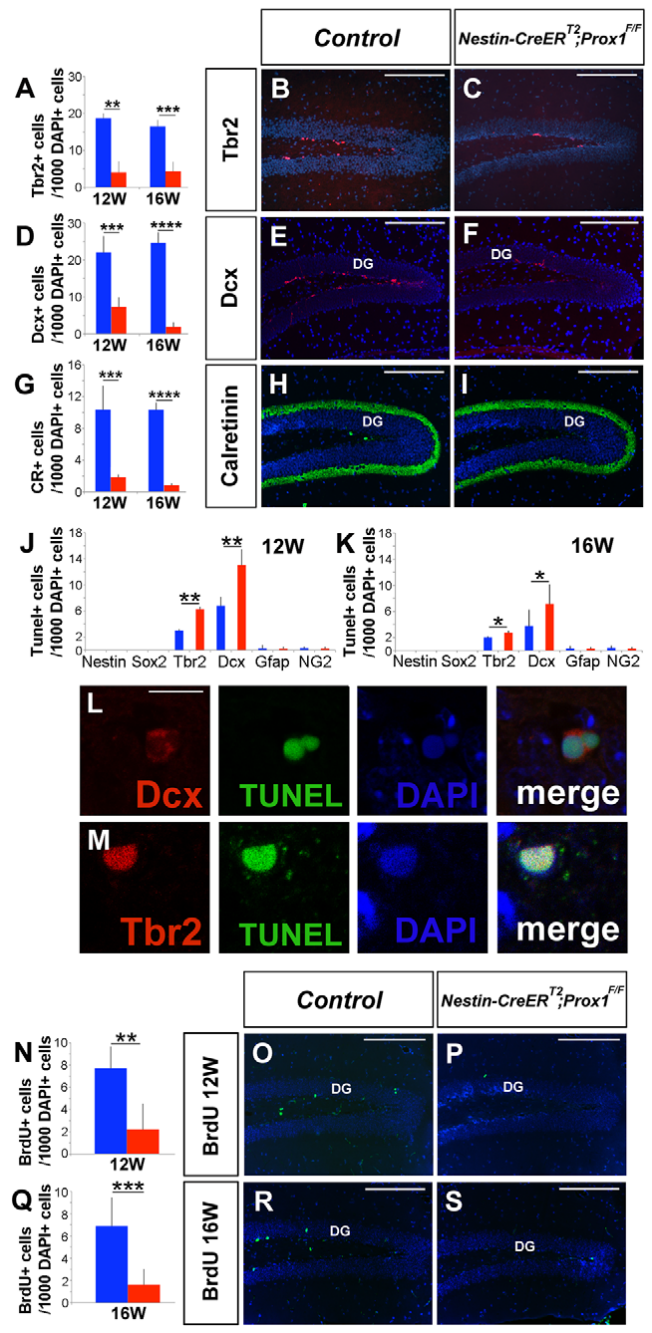
Prox1 is required during SGZ neurogenesis for the maintenance of intermediate progenitors. A reduced number of Tbr2^+^ intermediate progenitors (A–C) and of Dcx^+^ (D–F) and Calretinin^+^ (G–I) cells was observed in the SGZ of *Nestin-CreER^T2^;Prox1^F/F^* mice. An increase in the number of TUNEL^+^/Tbr2^+^ (J, M) and TUNEL^+^/Dcx^+^ (J, L) cells was observed in 12-wk-old and 16-wk-old (K) *Nestin-CreER^T2^;Prox1^F/F^* mice. As a consequence of the reduction in the number of intermediate progenitors, fewer BrdU^+^ cells are found in the DG of *Nestin-CreER^T2^;Prox1^F/F^* mice 4 (N–P) and 8 (Q–S) wk after the beginning of TM treatment. Data represent the mean number of positive cells per DG section ± SD. (*N* = 4 mice). Blue bars are controls. Red bars are TM-treated *Nestin CreER^T2^;Prox1^F/F^* brains. Paired *t* test. * *p*<0.1; ** *p*<0.01; *** *p*<0.001; **** *p*<0.0001. W, weeks. Scale bar: 100 µm. Scale bar in (L, M): 10 µm.

To determine whether the lack of intermediate progenitors in adult stages is a direct or indirect result of previously identified alterations in postnatal DG development, we treated 8-wk-old (adult) *Nestin-CreER^T2^*;*Prox1^F/F^* mice with TM 3 d/week (see Figure S3C for the details of the TM treatment). Four weeks after the initial TM induction, the number of Tbr2^+^ (Figure 7A–C) and Dcx^+^ (Figure 7D–F) cells was reduced in the SGZ of 12-wk-old *Nestin-CreER^T2^*;*Prox1^F/F^* mice. Accordingly, the number of Calretinin^+^ cells was also reduced (Figure 7G–I). Similar results were observed 8 wk after induction (in 16-wk-old animals) (Figure 7A,D,G).

To evaluate whether the lack of Prox1 activity leads to an increase in cell death, as it does during developmental stages, we performed double TUNEL immunohistochemistry in 12- and 16-wk-old mice after TM induction. At both time points, we observed an increased number of TUNEL^+^Tbr2^+^ (Figure 7J,K,M) and TUNEL^+^Dcx^+^ (Figure 7J–L) cells in the *Nestin-CreER^T2^*;*Prox1^F/F^* SGZ, a result indicating that Prox1 is required for the survival of Tbr2^+^ and Dcx^+^ cells.

To evaluate whether the lack of Tbr2^+^ intermediate progenitors and Dcx^+^ cells affects adult neurogenesis in the SGZ, we performed BrdU labeling 15 d prior to collection of the brains for analysis. Fewer BrdU^+^ newborn cells were observed in the SGZ of 12-wk-old (Figure 7N–P) and 16-wk-old (Figure 7Q–S) *Nestin-CreER^T2^*;*Prox1^F/F^* mice. This result suggests that the lack of Prox1 in the SGZ affects adult neurogenesis.

Next, to determine whether the defective neurogenesis in the SGZ is solely because of a reduction in the number of intermediate progenitors or it is also because of an alteration in immature neurons, we analyzed the ratio of Tbr2^+^:Calretinin^+^ cells in the SGZ of 12-wk-old and 16-wk-old *Nestin-CreER^T2^*;*Prox1^F/F^* mice. A consistent increase in the ratio of Tbr2^+^:Calretinin^+^ cells was observed in 12-wk-old (*N* = 3) (2.39±0.58:1) and 16-wk-old (*N* = 3) (4.39±1.92:1) TM-treated *Nestin-CreER^T2^*;*Prox1^F/F^* mice (12-wk-old control: 1.77±0.34:1; 16-wk-old control: 1.51±0.17:1; *p* value for both cases was *p*<0.1). This result suggests that granule cell maturation is also affected by the lack of Prox1 in the SGZ during adult neurogenesis.

We next examined whether the induction of neurogenesis rescues the reduction in the number of progenitor cells observed in the SGZ of adult *Nestin-CreER^T2^*;*Prox1^F/F^* mice. At 12 wk of age, TM-induced and control mice were treated with kainic acid (KA), a compound that induces adult hippocampal neurogenesis (see Figure S3D for the details of the TM/KA treatment) [39],[40]. Eight days after KA administration, the brains of control mice treated with TM and KA or only with KA showed an increased number of Tbr2^+^ (Figure S8A,B), Dcx^+^ (Figure S8D,E), or Calretinin^+^ (Figure S8G,H) cells in the SGZ. However, similar to that in the TM-treated *Nestin-CreER^T2^*;*Prox1^F/F^* mice, the SGZ of *Nestin-CreER^T2^*;*Prox1^F/F^* mice treated with TM and KA had fewer Tbr2^+^ intermediate progenitors (Figure S8A,C) and fewer Dcx^+^ (Figure S8D,F) and Calretinin^+^ (Figure S8G,I) cells. These results show that KA-mediated induction of neurogenesis cannot rescue the reduction in the number of Tbr2^+^, Dcx^+^, or Calretinin^+^ cells in the SGZ of TM-treated adult *Nestin-CreER^T2^*;*Prox1^F/F^* mice.

### Lack of Intermediate Progenitors Results in Defective Self-Maintenance of Adult Neural Stem Cells

It has been proposed that upon their differentiation, intermediate progenitors trigger a feedback mechanism necessary to stop NSC differentiation and support NSC maintenance [13],[15]. Therefore, the lack of intermediate progenitors observed in the SGZ of *Nestin-CreER^T2^*;*Prox1^F/F^* mice may impinge on this feedback mechanism and ultimately affect the number of adult NSCs produced in this region.

**Figure 8:**
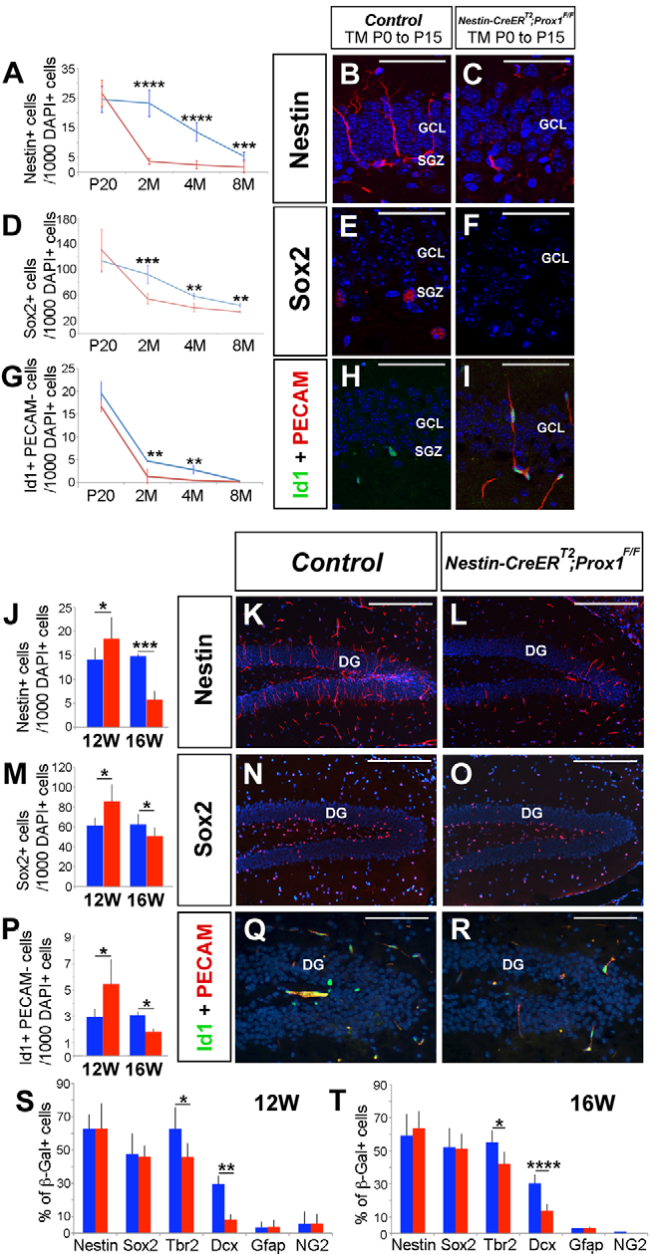
Adult NSCs in the SGZ do not self-maintain in the absence of intermediate progenitors. Nestin (B, C), Sox2 (E, F), and Id1/PECAM (H, I) immunostaining of the subgranular zone (SGZ) of 4-mo-old controls (B, E, H) and *Nestin-CreER^T2^;Prox1^F/F^* (C, F, I) mice treated with TM from P0 to P15. Graphs compare the number of Nestin^+^ (A), Sox2^+^ (D), and Id1^+^PECAM^−^ (G) cells in P20, 2-mo-old, 4-mo-old, and 8-mo-old control (blue) and *Nestin-CreER^T2^;Prox1^F/F^* (red) mice treated with TM from P0 to P15. At P20, the number of Nestin^+^, Sox2^+^, or Id1^+^PECAM^−^ NSCs is similar in controls and *Nestin-CreER^T2^;Prox1^F/F^* brains (A, D, G). However, after P20, the number of Nestin^+^ (A–C), Sox2^+^ (D–F), or Id1^+^PECAM^−^ (G–I) cells is reduced in *Nestin-CreER^T2^;Prox1^F/F^* mice. Graphs compare the number of Nestin^+^ (J), Sox2^+^ (M), and Id1^+^ PECAM^−^ (P) cells in 12-wk-old and 16-wk-old control (blue) and *Nestin-CreER^T2^;Prox1^F/F^* (red) mice treated with TM for 4 wk starting at 8 wk of age. The number of Nestin^+^, Sox2^+^, and Id1^+^ PECAM^−^ adult NSCs was increased in 12-wk-old conditional mutant brains (J, M, P). At 16 wk, the number of Nestin^+^ (J, L), Sox2^+^ (M, O), and Id1^+^ PECAM^-^ (P, R) cells is reduced in the SGZ of conditional-mutant mice. The percentage of Nestin^+^/b-Gal^+^ and Sox2^+^/b-Gal^+^ cells is similar in 12-wk-old (S) and 16-wk-old (T) control (blue) and *Nestin-CreER^T2^;Prox1^F/F^* (red) brains that carry the *ROSA* allele. However, the percentage of Tbr2^+^/b-Gal^+^ and Dcx^+^/b-Gal^+^ cells is reduced at both stages in the *Nestin-CreER^T2^;Prox1^F/F^*;*ROSA* SGZ (S, T). Data represent the mean number of positive cells per DG section ± SD. *N* = 4 brains. Paired *t* test. * *p*<0.1; ** *p*<0.01; *** *p*<0.001; **** *p*<0.0001. GCL, Ganglion cell layer. Scale bar in (B, C, E, F, H, I): 25 µm. Scale bar in (K, L, N, O): 100 µm. M, month; W, weeks. Scale bar in (Q, R): 50 µm.

To assess whether the reduced number of intermediate progenitors in the SGZ of *Nestin-CreER^T2^*;*Prox1^F/F^* mice treated postnatally with TM affected that of NSCs, we compared the number of Type-I NSCs in wild-type and Prox1 conditional-mutant brains. We detected no difference in the number of Nestin^+^, Sox2^+^, or Id1^+^ adult NSCs at P20 (Figure 8A,D,G). However, we unexpectedly observed fewer Nestin^+^, Sox2^+^, or Id1^+^ NSCs in the SGZ of *Nestin-CreER^T2^*;*Prox1^F/F^* mice older than 2 mo (Figure 8A–I). We also observed a similar reduction in the number of Nestin^+^ Gfap^+^ Blbp^+^ NSCs in the SGZ of 4-mo-old *Nestin-CreER^T2^*;*Prox1^F/F^* mice (Figure S9). This result argued that the absence of intermediate progenitors observed in the SGZ of *Nestin-CreER^T2^*;*Prox1^F/F^* brains leads to a non-cell autonomous reduction in the number of adult NSCs.

To determine whether defective maintenance of adult NSCs is a direct result of the lack of intermediate progenitors or an indirect result because of alterations in DG development, we treated 8-wk-old control and *Nestin-CreER^T2^*;*Prox1^F/F^* mice with TM (see Figure S3C for the details of the TM treatment) and counted the number of adult NSCs. Four weeks after the beginning of the treatment, the number of Nestin^+^, Sox2^+^, or Id1^+^ adult NSCs was higher in the SGZ of *Nestin-CreER^T2^*;*Prox1^F/F^* mice than in wild-type littermates (Figure 8J,M,P); however, 8 wk after TM treatment, we detected fewer Nestin^+^, Sox2^+^, or Id1^+^ adult NSCs in the SGZ of *Nestin-CreER^T2^*;*Prox1^F/F^* mice (Figure 8J–R).

To examine whether Prox1 has a direct role in this defective maintenance of adult NSCs, we generated *Nestin-CreER^T2^*;*Prox1^F/F^;R26R* mice and treated them with TM (3 d/wk) starting at 8 wk of age (see Figure S3C for the details of the TM treatment). After 4 wk of treatment, the ratios of Nestin^+^β-gal^+^ cells and Sox2^+^β-gal^+^ cells were the same in wild-type and *Prox1* conditional-mutant mice (Figure 8S). This result indicates that the absence of Prox1 did not directly affect the adult NSC population. Similar results were seen in mice analyzed 8 wk after the beginning of TM administration (Figure 8T). Only the Tbr2^+^β-gal^+^ and the Dcx^+^β-gal^+^ cell populations were significantly reduced in the SGZ of *Nestin-CreER^T2^*,*Prox1^F/F^;R26R* mice at 4 and 8 wk after the beginning of TM treatment (Figure 8S,T). These results, together with the absence of increased apoptosis in adult NSCs (Figure 7J,K), suggest that the defective maintenance of adult NSCs in the mutant SGZ is indirectly caused by the absence of intermediate progenitors.

**Figure 9:**
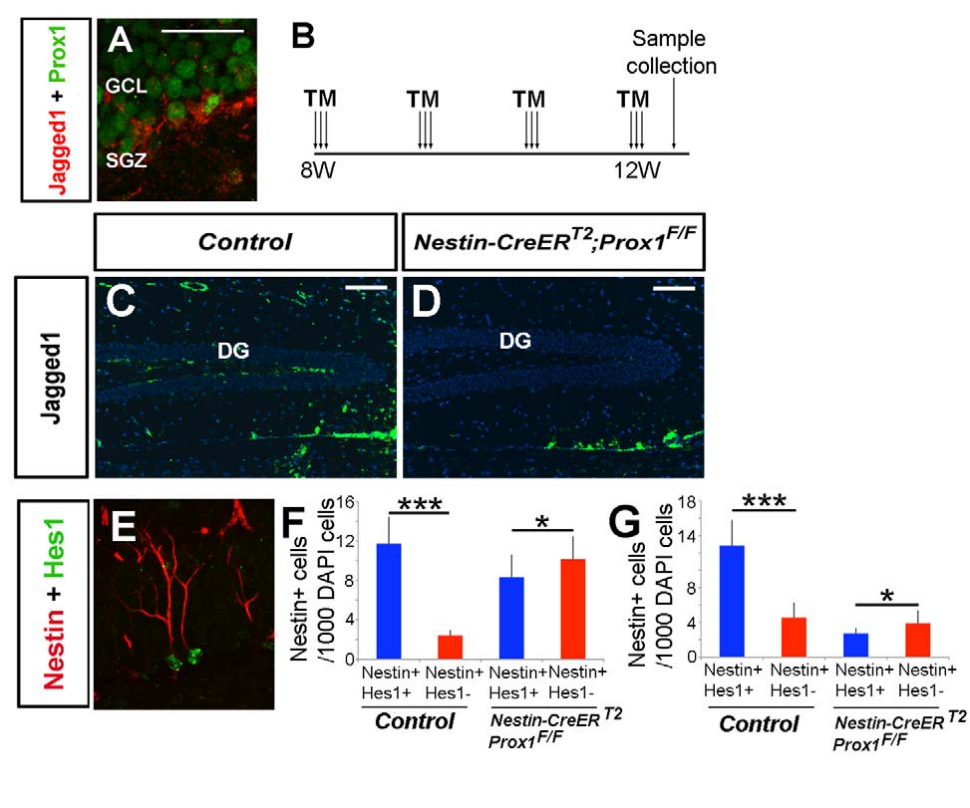
Lack of intermediate progenitors disrupts *Notch* signaling in the SGZ of *Nestin-CreER^T2^;Prox1^F/F^* mice. (A) Prox1^+^/Jagged1^+^ cells in the SGZ of adult mice. (B) TM administration schedule. (C, D) Jagged1^+^ cells were not detected in 12-wk-old *Nestin-CreER^T2^;Prox1^F/F^* mice after 4 wk of TM treatment. (E) Nestin^+^Hes1^+^ cells are present in the SGZ of control and *Nestin-CreER^T2^;Prox1^F/F^* mice. However, the number of double positive cells is reduced in TM-treated *Nestin-CreER^T2^;Prox1^F/F^* in 12-wk-old (F) and 16-wk-old mice (G). Data represent the mean number of positive cells per DG section ± SD. *N* = 3 brains. Paired *t* test. *** *p*<0.001. Scale bar in (A, E): 25 µm. Scale bar in (C, D): 100 µm.

We next investigated the possible mechanisms of this intermediate progenitor-dependent control of adult neurogenesis in the SGZ. In the developing telencephalon [41] and in the SGZ [42], Notch signaling is necessary for NSC maintenance, and the expression of Jagged1, a Notch receptor ligand, is restricted to intermediate progenitors in the SGZ [42]. We found a reduced number of Jagged1^+^ cells in the SGZ of *Nestin-CreER^T2^*;*Prox1^F/F^* mice treated with TM starting at 8 wk of age and analyzed 4 wk later (Figure 9C,D). To examine the relationship between the number of Nestin^+^ Type-I cells in the SGZ and active Notch signaling, we performed Hes1 immunohistochemistry. In the SGZ of control mice, only 17% of the Type-I cells were Nestin^+^Hes1^–^ (Figure 9F); however, in the *Nestin-CreER^T2^*;*Prox1^F/F^* mice, this number increased to 55% (Figure 9F). The number was also higher in the SGZ of *Nestin-CreER^T2^*;*Prox1^F/F^* mice (59%) when compared with wild-type controls (25%) at 16 wk of age (Figure 9G). A reduced number of *Hes5*-expressing cells was also observed in the SGZ of *Nestin-CreER^T2^*;*Prox1^F/F^* mice at 16 wk of age (Figure S10). These results show that in the absence of intermediate progenitors, the proportion of adult NSCs with active Notch signaling at 12 and 16 wk is reduced. This mechanism may explain why the self-maintenance of adult NSCs is eventually overruled at 16 wk of age.

### Ectopic Expression of Prox1 in NSCs Induces Their Premature Differentiation

**Figure 10:**
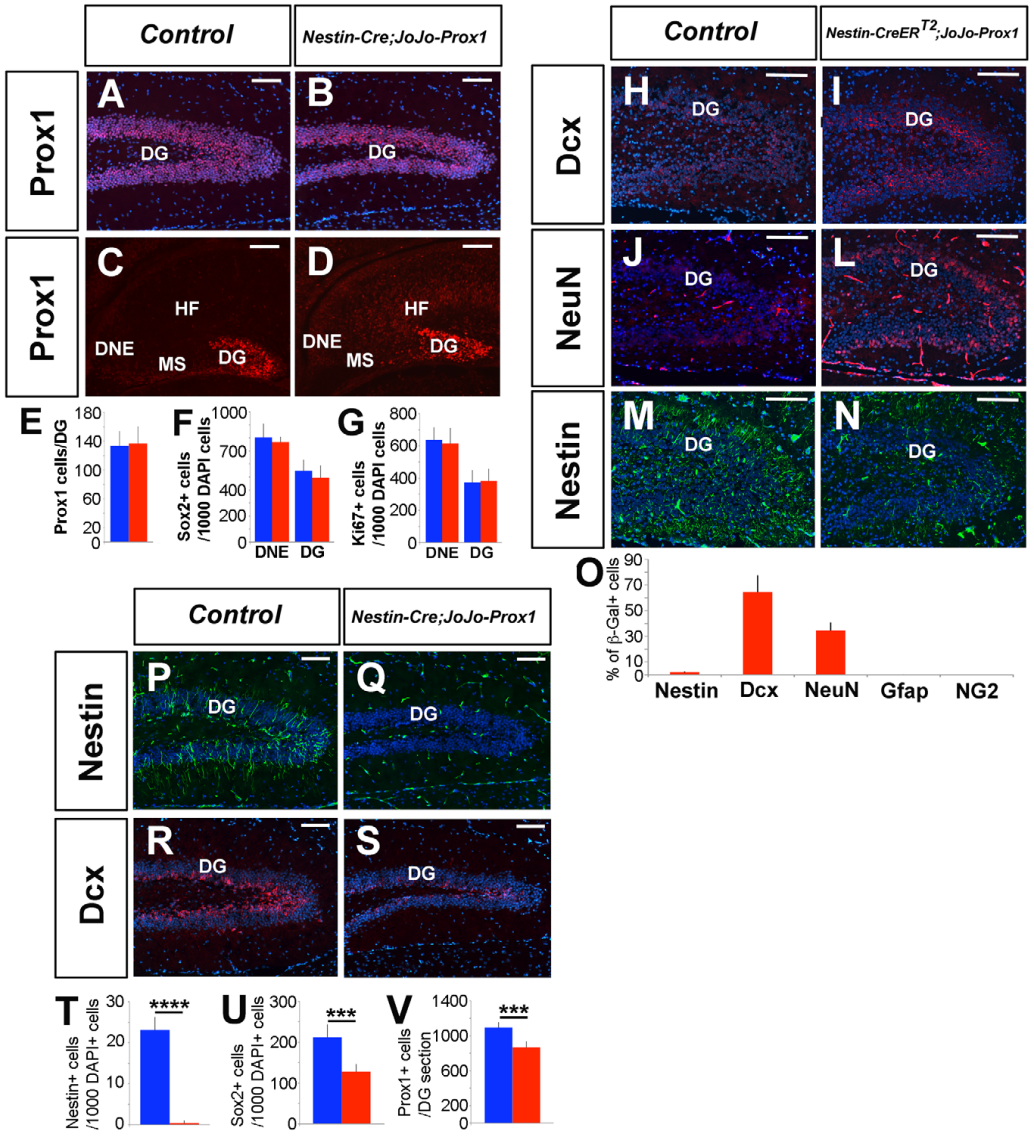
Prox1 ectopic-expression promotes NSC premature differentiation. Anti-C-Prox1 staining (A, control) shows a reduced dentate gyrus (DG) in P30 *Nestin-Cre;JoJo(Prox1)* mice (B, V). There is no difference in the number of Prox1^+^ cells in the DG region of control (C, E) and *Nestin-Cre;JoJo(Prox1)* (D, E) at E16.5. There are no differences either in number of Sox2^+^ (F) or Ki67^+^ (G) cells in the dentate neuroepithelium (DNE) and the DG at E16.5. At P8, there is an increase in the number of Dcx^+^ (H, I) and NeuN^+^ (J, L) cells in TM-treated *Nestin-CreER^T2^;Prox1^F/F^* brains. However, the number of Nestin^+^ (M, N) cells is reduced at this stage. (O) Cell fate analysis of the Prox1 ectopic-expressing cells, showing that they predominantly differentiate towards a neuronal lineage. The number of Nestin^+^ adult NSCs (P, Q, T) and Dcx^+^ intermediate progenitors (R, S, U) is reduced in the SGZ of P30 *Nestin-Cre;JoJo-Prox1* animals. Data represent the mean number of positive cells per DG section ± SD. *N* = 3 brains. Paired *t* test. *** *p*<0.001. Blue bars are controls. Red bars are *Nestin-Cre;JoJo-Prox1.* MS, Migratory stream; HF, Hippocampal field. Scale bar: 100 µm.

Our results showed that Prox1 is necessary for granule cell differentiation and intermediate progenitor maintenance. Therefore, we next determined whether Prox1 activity is sufficient to induce granule cell differentiation. To do this, we generated a new mouse transgenic line in which *Prox1* expression was under the control of the ubiquitous CMV promoter (*CMV-CAG-loxP-eGFP-Stop-loxP-Prox1-Ires-β Gal; JoJo-Prox1* for brevity) [43]. Crossing this line with any available Cre strain will lead to the release of the Stop-signal cassette and the transcription of *Prox1* in a tissue-specific manner. We used this novel strain to generate adult *Nestin-Cre;JoJo-Prox1* mice that ectopically expressed *Prox1* in several brain regions (Figure S11). Importantly, we found that the number of granule cells was reduced in the DG of these mice (Figure 10A,B,V).

*Prox1* was ectopically expressed in the ventricular, subventricular, and mantle zones of several brain regions of *Nestin-Cre;JoJo-Prox1* embryos (Figure S11), including the hippocampal neuroepithelium and the hippocampal field (Figure 10D). However, we observed no change in the number of Prox1^+^ (Figure 10E), Sox2^+^ (Figure 10F), or Ki67^+^ cells (Figure 10G) in the DNE or DG. These results suggest that ectopic expression of Prox1 in embryonic *Nestin*-expressing neuroepithelium is not sufficient to affect embryonic NSC differentiation.

To perform a similar analysis during postnatal stages, we administered TM to *Nestin-CreER^T2^*;*JoJo-Prox1* pups daily starting at P0 (see Figure S3B for the details of the TM treatment). At P8, the DG of *Nestin-CreER^T2^*;*JoJo-Prox1* pups exhibited an increased number of Dcx^+^ (Figure 10I) and NeuN^+^ (Figure 10L) cells and a reduced number of Nestin^+^ cells (Figure 10N). At this stage, proliferation was already reduced in the DG of *Nestin-CreER^T2^*;*JoJo-Prox1* mice, as shown by Ki67 staining (Figure S12G).

To determine the fate of cells that ectopically expressed Prox1, we performed double immunohistochemistry using antibodies against β-gal and Nestin, Dcx, NeuN, Gfap, or NG2 (Figure S12I–M). We determined that 2.11%±0.38% of the β-gal^+^ cells in the DG of *Nestin-CreER^T2^*;*JoJo-Prox1* mice were Nestin^+^; 64.57%±12.99% were Dcx^+^; 34.52%±6.32% were NeuN^+^; and less than 0.1% were Gfap^+^ or NG2^+^ (Figure 10O). No increase in the number of TUNEL^+^ cells was observed in the DG of these mice (Figure S12H). These results suggest that there is premature neuronal differentiation in the DG of *Nestin-CreER^T2^*;*JoJo-Prox1* mice during postnatal stages of brain development.

Finally, we addressed the consequences of ectopic expression of *Prox1* in the SGZ during adult neurogenesis. Analysis of the brains of P30 *Nestin-CreER^T2^*;*JoJo-Prox1* mice revealed the presence of Nestin^+^;Prox1^+^ adult NSCs (Figure S12N). As a consequence of *Prox1* ectopic expression, the numbers of Nestin^+^ (Figure 10Q,T) and Sox2^+^ (Figure S12P) cells were reduced at this stage. Accordingly, the numbers of Dcx^+^ (Figure 10S,U) and Calretinin^+^ (Figure S12R) intermediate progenitors were also reduced. These results indicate that the ectopic expression of *Prox1* depleted the adult NSC population.

## Discussion

In this article, we report for the first time the functional role for *Prox1* in mammalian brain development. We determined that in the mouse, *Prox1* is required for the maturation of granule cells during DG development. *Prox1* is expressed through all the stages of DG formation; therefore, it is possible that the defective granule cell maturation observed in *Nestin-Cre;Prox1^F/F^* mice is an indirect consequence of the earlier absence of Prox1 at the intermediate progenitor level. However, it is also possible that Prox1 plays additional functional roles during granule cell formation. Moreover, the granule cells in the DG are one of the few types of brain cells that express *Prox1* throughout adulthood. This suggests that Prox1 might be necessary not only for the maturation of granule cells but also for the regulation of other aspects of granule cell function. NeuroD1 is also required for granule cell maturation [15],[29],[30]. During embryogenesis and in the absence of Prox1 or NeuroD1, mutant granule cells express some neuronal markers but fail to fully differentiate and undergo apoptosis [15]. As a consequence, *Nestin-Cre;Prox1^F/F^* and *NeuroD1^–/–^* mice have very few granule cells (Figure 1) [15],[30]. Few Prox1^+^ granule cells are present in the DG of *NeuroD1^–/–^* mice [15], and NeuroD1 is expressed in the DG of *Prox1*-conditional mutants. These results suggest that although NeuroD1 and Prox1 might control similar or parallel pathways of granule differentiation, they might not necessarily induce each other's expression.

We also showed that during adult neurogenesis, Prox1 is necessary for the survival of Tbr2^+^ intermediate progenitors in the SGZ. In this case and similarly to DG development, our data suggest that the lack of Prox1 results in defects in granule cell maturation that could also be an indirect consequence of the earlier lack of Prox1 at the progenitor level. Nevertheless, the lack of Prox1 leads to an increase in apoptosis in Tbr^+^ and Dcx^+^ cells and the absence of adult neurogenesis. Also in this case, similar results have been reported for *NeuroD1*-mutant mice during adult neurogenesis [44],[45].

Our results provide the strongest evidence so far about a feedback mechanism involved in the regulation of adult neurogenesis and progenitor cell numbers in the adult SGZ. We also provide evidence supporting the proposal that Tbr2^+^ intermediate progenitors and Dcx^+^ cells are required to maintain the adult NSC population in the SGZ niche. We showed that in the absence of Tbr2^+^ and Dcx^+^ cells and as this feedback regulation becomes defective, adult NSCs continue generating new progeny such that the adult NSC population in the SGZ of *Prox1*-conditional mutants transiently expands but is ultimately depleted. We do not yet know whether this depletion of NSCs is due to their premature differentiation or their exhaustion resulting from increased proliferation [46].

How is this feedback mechanism regulated? We showed that in the SGZ of *Prox1* conditional-mutant mice the lack of intermediate progenitors leads to a reduction in Jagged1 expression. Moreover, we determined that in these conditional-mutant mice the lack of intermediate progenitors leads to the absence of active Notch signaling in adult NSCs; active Notch signaling is necessary for the maintenance of adult stem cells in the brain, bone marrow, and gut [42],[47]–[51]. Previous work has shown that during embryonic development, the activity of *mind bomb homolog 1* (*Mib1*) is required for Jagged and Delta-like-mediated Notch signaling. In the absence of Mib1, Notch signaling is not activated in radial glia cells, which results in their premature differentiation [41],[52]. In the SGZ, the absence of Notch1 is sufficient to induce neuronal differentiation and reduce the proportion of adult NSCs and intermediate progenitors, which suggests that this receptor is involved in NSC self-renewal in the SGZ [42]. Therefore, the role of other Notch receptors during adult neurogenesis could be either redundant or restricted to a subpopulation of cells in the SGZ [42],[53].

Together, these and others results [42] suggest that in the SGZ, the *Notch1/Jagged1* pathway may regulate NSC self-maintenance. However, we cannot exclude the possibility that Notch activation in adult NSCs is also mediated by other Notch ligands expressed by other cell types (e.g., vascular endothelial cells) [54]. Also, we cannot rule out the possibility that other signaling molecules or trophic factors produced by *Prox1*-expressing intermediate progenitors may be involved in the feedback mechanism that controls adult neurogenesis in the SGZ.

In summary, we have provided evidence demonstrating that Prox1 plays a key role as a neurogenic factor during granule cell formation. In the absence of Prox1, granule cell maturation is affected at later stages of differentiation; ectopic expression of *Prox1* in the brain is sufficient to overrule the mechanisms that control NSC self-maintenance and induce premature differentiation in the SGZ during postnatal stages of brain development and adult neurogenesis.

## Materials and Methods

### Mice

*Prox1^F/F^* [21] mice, *Nestin-Cre* [22] mice, and *Nestin-CreER^T2^* [28] mice were previously described. The *JoJo-Prox1* construct was generated by the introduction of a 2.2 kb *Prox1* cDNA into a *CMV-CAG-loxP-eGFP-Stop-loxP-IRES-βGal* expression vector [43]. Mice were kept in the NMRI background. TM (Sigma) was dissolved in safflower oil at 20 mg/ml. For prenatal induction, time-mated females were treated with TM by gavage at E16.5 and embryos were harvested at E18.5. To induce Cre recombination postnatally, pups were fed TM (4 mg/20 g body weight) daily from P0 until the collection day. As a consequence of postnatal TM administration, body weight was reduced in TM-treated versus non-TM-treated pups. This reduction was temporary; 2-mo-old postnatally TM-treated *Nestin-CreER^T2^;Prox1^F/+^* and *Nestin-CreER^T2^;Prox1^F/F^* mice had a similar weight than non-TM-treated animals. In adults (8-wk-old), TM (4 mg/20 g body weight) was administered by gavage 3 times/wk for 4 wk. No obvious weight differences were observed in the TM-treated animals at the time of sample collection. Genotypes were determined by PCR analysis. *Nestin-Cre;Prox1^F/+^*, *Nestin-CreER^T2^;Prox1^F/+^*, and *JoJo-Prox1* mice were used as controls.

### Immunohistochemistry

For Hes1 staining during adult stages, brains were perfused with 4% PFA and embedded in paraffin. Antigen retrieval was performed for 7 min at 105°C in a decloaking chamber with Antigen Retrieval Solution (Dako). The ABC Kit (Vector Laboratories) and TSA Fluorescein System (Perkin Elmer) were used for signal amplification. Jagged1 staining at adult stages was performed on brains perfused with 4% PFA and cryoprotected in 30% sucrose. Signal amplification was obtained with the ABC Kit and the TSA Fluorescein System. Immunohistochemistry for the rest of the antibodies was performed as described [17]. The following antibodies and dilutions were used: rabbit anti-C-Prox1 (1∶1000; Millipore), guinea pig anti-C-Prox1 (1∶100; our own), rabbit anti-N-Prox1 (1∶1000; a gift from B. Sosa-Pineda), goat anti-N-Prox1 (1∶100; R&D systems), goat anti-Nestin (1∶100; R&D systems), rabbit anti Id-1 (1∶200; Biocheck), rat anti-PECAM (1∶500; Pharmingen), mouse anti-Gfap (1∶500; Sigma), rabbit anti Blbp (1∶200; Chemicon), rabbit anti-Sox2 (1∶1000; Milipore), rabbit anti-Sox2 (1∶500; Invitrogen), mouse anti-Sox2 (1∶50, Milipore), mouse anti-Ascl1 (1∶100; Milipore), mouse anti-βTubIII (1∶500; BabCO), rabbit anti-NeuroD1 (1∶500; Chemicon), rabbit anti-Dcx (1∶100; Abcam), rabbit anti-Dcx (1∶1000; Chemicon), rabbit anti-Dcx (1∶500; Cell Signaling), rabbit anti-Calretinin (1∶5000; Millipore), mouse anti-NeuN (1∶100; Millipore), rabbit anti-active caspase-3 (1∶100; BD-Pharmingen), rabbit anti-Hes1 (1∶50, Santa Cruz), goat anti-Jagged1 (1∶50, Santa Cruz), rabbit anti-GFP (1∶1000; Molecular Probes), rabbit anti-bGal (1∶1000, ICN), and chicken anti-bGal (1∶1000, Abcam). The following secondary antibodies were used: anti-rabbit, anti-mouse, anti-guinea pig, anti-chicken, or anti-goat Alexa 488, Alexa 594 (Molecular Probes), Cy3, or Cy5 (Jackson Immunoresearch). Low-magnification images were obtained with a Leica MZFLIII stereomicroscope equipped with a Hanamatsu C5810 camera and a Zeiss Axiovert 1.0 microscope equipped with a ProgRes C14 camera. The remaining images were obtained with a Leica SP1 confocal microscope or a Zeiss LSM 510 NLO Meta confocal microscope.

### In Situ Hybridization

In situ hybridization of sections was performed as previously described [55]. The following probes were obtained from: *Ngn2* (Q. Ma), *Wnt3a* (A. McMahon), *Lhx2* (H. Westphal), *Notch1* (G. Weinmaster), *Lef1* (G. Kardon). Double–in situ hybridization/immunohistochemistry was performed as described [17].

### TUNEL and Proliferation Assays

TUNEL assay was performed on tissue sections as previously described [56]. For proliferation assays at embryonic stages, time-mated female mice were injected with BrdU (100 µg/g body weight, intraperitoneally), and embryos were harvested 1 h later. Embryos were fixed o/n in 4% PFA and cryoprotected in 30% sucrose. For proliferation assays at early postnatal stages, P5 and P10 pups were injected with BrdU (100 µg/g body weight, intraperitoneally) 1 h before harvest. Brains were perfused with 4%PFA and cryoprotected in 30% sucrose. For proliferation assays at adult stages, animals were injected with BrdU (100 µg/g body weight, intraperitoneally) 15 d before harvest. Brains were perfused in 4% PFA and cryoprotected in 30% sucrose. BrdU incorporation was exposed after 20-min treatment in 2N HCl. Mouse anti-BrdU (1∶10; BD Biosciences) antibody was used. Sections were counterstained with DAPI.

### Neurosphere Cultures and Immunocytochemistry

Neurosphere cultures were established as described, with modifications [57]. Briefly, E16.5 hippocampi were dissected, disaggregated in trypsin, and maintained in culture in neurosphere culture medium (Neurobasal medium with GlutaMAX, Pen/Strep, B27, and N2) (Gibco) supplemented with 20 ng/ml EGF (Upstate) and 20 ng/ml FGF (Millipore). After the fourth passage, neurospheres were differentiated for 4 d in neurosphere culture medium with 10% fetal calf serum (FCS) without supplements, in Lab-Tek II CC^2^ chamber slides (Nunc). Cells were fixed in 2% PFA for 15 min at room temperature (RT). Cells were blocked in 10% FCS for 30 min at RT and incubated with the appropriate primary and secondary antibodies (see above) in 2% FCS for 2 h. For the transfection of the neurospheres, a Mouse NSC Nucleofector® Kit (Amaxa) was used according to the manufacturer's specifications. For *Prox1* expression, a *Prox1* cDNA was cloned in an expression plasmid under the PGK promoter.

### Kainic Acid Assay

Adult neurogenesis was induced by KA as described [39] with minor modifications. Briefly, TM (4 mg/20 g body weight) was administered by gavage 3 times/wk for 4 wk to 8-wk-old *Nestin-CreER;Prox1^F/F^* and *Nestin-CreER;Prox1^F/+^* mice. At the end of TM treatment, animals were injected intraperitoneally with KA (30 mg/kg body weight; Sigma) dissolved in PBS. Approximately 40 min after KA injection, all mice displayed status epilepticus for 2 to 3 h. Approximately 15% of control and conditional mutant mice died 12 h after KA administration. Eight days after KA injection, the surviving animals were euthanized and their brains perfused with 4% PFA and cryoprotected in 30% sucrose.

## Supporting Information

Figure S1 **Detection of Δ-Prox1 cells in** ***Prox1*** **conditional mutant mice.** (A, B) Schematic representation of the different Prox1 alleles described in this article and the recognition domains of the anti-C-Prox1 and anti-N-Prox1 antibodies. (A) In the *Prox1* floxed allele, loxP sites flank part of the prospero domain (purple) and the homeodomain (green). (B) Following Cre-mediated excision (Δ form), only a nonfunctional N-terminal part of Prox1 remains, without most of the prospero domain and without the entire homeodomain. Anti-N-Prox1 antibodies recognize both the wild-type and Δ forms of the Prox1 protein, but anti-C-Prox1 antibodies recognize only the wild-type full-length protein. For more details, see Hervey et al. (2005). (C–F) Coronal sections of control (C, E) and *Nestin-Cre;Prox1^F/F^* (D, F) dentate gyrus at different developmental stages show the presence of the Δ-Prox1^+^ cell population in the conditional-mutant mice. Sections were immunolabeled with antibodies against N-Prox1^+^ (red) and C-Prox1^+^ (green).
(0.64 MB TIF)

Figure S2 **N-Prox1^+^ cells migrate from the dentate neuroepithelium to the region of the dentate gyrus in** ***Nestin-Cre;Prox1^F/F^*** **mice.** (A, B) *Wnt3a* is expressed in the fimbria neuroepithelium (FNE) (arrows) of E16.5 control and *Nestin-Cre;Prox1^F/F^* brains. (C, D) In response to Wnt3a signaling from the FNE, *Lef1* is expressed in N-Prox1^+^ migrating cells (arrows) in E16.5 control and mutant embryos. The radial glia scaffolding is normal in the *Nestin-Cre;Prox1^F/F^* dentate gyrus (DG), as shown by Gfap (E, F) and Nestin (G, H) immunostaining. DNE, Dentate neuroepithelium; MS, Migratory stream. Scale bar in (M–T): 50 µm.
(6.42 MB TIF)

Figure S3 **Schematic representation of the TM administration protocol.** (A) For prenatal induction, TM was administered to time-mated female mice at E16.5 and embryos were harvested at E18.5. (B) For postnatal induction, pups were fed daily with TM until collection day or P15. (C) For adult induction, 8-wk-old mice were treated with TM for 4 wk, 3 d a week. Samples were collected 4 wk or 8 wk after the beginning of TM treatment. (D) At the end of the TM treatment, kainic acid was administered to control and conditional mutant mice. The samples were collected 8 d after KA administration.
(0.28 MB TIF)

Figure S4 **Prox1 is necessary for neuronal differentiation in vitro.** (A–C) Neurospheres generated from the hippocampus of E16.5 control and *Nestin-Cre;Prox1^F/F^* mice are capable of producing βTub-III^+^ neurons (*N* = 3 experiments; 175 control and 135 *Nestin-Cre;Prox1^F/F^* neurospheres were analyzed). (D–F) However, most of the neurospheres obtained from *Nestin-Cre;Prox1^F/F^* mice failed to generate Dcx^+^ cells (*N* = 3 experiments; 216 control and 190 *Nestin-Cre;Prox1^F/F^* neurospheres were analyzed). (G, H) 3 d after co-transfection with GFP- and Prox1-expressing plasmids, around 20% of the *Nestin-Cre;Prox1^F/F^* GFP^+^ neurospheres generated Dcx^+^ neurons (*N* = 3 experiments; a total of 76 *Nestin-Cre;Prox1^F/F^* GFP^+^ neurospheres and 73 GFP^+^ *Nestin-Cre;Prox1^F/F^* neurospheres transfected with Prox1 were analyzed). Data represent the mean number of the percentage of neurospheres per experiment ± SD. Paired *t* test. *** *p*<0.001; **** *p*<0.0001. Scale bar in (A, B, D): 50 µm. Scale bar in (E): 100 µm. Scale bar in (G): 25 µm.
(1.52 MB TIF)

Figure S5 **The number of intermediate progenitors and maturing granule cells is reduced in the dentate gyrus of P5** ***Nestin-Cre;Prox1^F/F^*** **pups treated with TM from P0 to P5.** (A–E) Sox2^+^ Prox1^+^ cells are not detected in the wild-type dentate gyrus (DG) at P10. (F) C-Prox1^+^ cells are present in the control DG and the hilus (H) at P5. (G) TM administration from P0 to P5 reduces the number of C-Prox1^+^ cells in the hilus of P5 *Nestin-CreER^T2^;Prox1^F/F^* pups. However, no alterations in the number of Sox2^+^ (H–J) or Id1^+^ PECAM^−^ (K–M) NSCs were detected in *Nestin-CreER^T2^;Prox1^F/F^* mutant brains. (N–P) Following a 1-h pulse, BrdU immunostaining revealed a small reduction in proliferation in the DG of *Nestin-CreER^T2^;Prox1^F/F^* brains. (Q–S) As shown by Tbr2 staining, the number of intermediate progenitors is reduced in the DG of the conditional mutant mice at this stage. (T, U) The number of Dcx^+^ cells is also reduced in the DG of *Nestin-CreER^T2^;Prox1^F/F^* pups at P5. An increase in the number of TUNEL^+^ cells (V–Y) is observed in the DG of *Nestin-CreER^T2^;Prox1^F/F^* brains at P5. Data represent the mean number of positive cells per DG section ± SD (*N* = 3 mice). Paired *t* test. * *p*<0.1; **** *p*<0.0001. GCL, granule cell layer; SGZ, Subgranular zone. Scale bar: 100 µm.
(2.97 MB TIF)

Figure S6 **The dentate gyrus of TM-treated** ***Nestin-CreER^T2^;Prox1^F/F^*** **pups exhibits an increased number of active caspase-3^+^ cells at P5 and P10.** The number of active caspase-3^+^ cells is increased in the dentate gyrus of P5 (A) and P10 (B) TM-treated *Nestin-CreER^T2^;Prox1^F/F^* pups. Data represent the mean number of positive cells per DG section ± SD (*N* = 3 mice). Paired *t* test. ** *p*<0.01; **** *p*<0.0001.
(0.12 MB TIF)

Figure S7 **Dcx^+^ cells remaining in TM-treated** ***Nestin-CreER^T2^;Prox1^F/F^*** **mice are those that have escaped Cre-mediated deletion.** Double C-Prox1/Dcx immunostaining shows that in 2-mo-old *Nestin-CreER^T2^;Prox1^F/F^* mice treated with TM from P0 to P15 the remaining Dcx^+^ cells were also C-Prox1^+^.
(1.97 MB TIF)

Figure S8 **Kainic Acid do not induce adult neurogenesis in the SGZ of TM-treated** ***Nestin-CreER^T2^;Prox1^F/F^*** **mice.** (A, B) The number of Tbr2 intermediate progenitors is increased in the dentate gyrus (DG) of control mice treated with TM or with TM and Kainic Acid (KA). However, KA is not able to increase the number of Tbr2^+^ cells in 12-wk-old TM-treated *Nestin-CreER^T2^;Prox1^F/F^* mice (A, C). Similar results were observed with Dcx^+^ (D–F) and Calretinin^+^ (G–I) cells. Data represent the mean number of positive cells per DG section ± SD (*N* = 3 mice). Paired *t* test. * *p*<0.1; ** *p*<0.01; *** *p*<0.001. Scale bar: 100 µm.
(3.77 MB TIF)

Figure S9 **Analysis of the NSC population in TM-treated** ***Nestin-CreER^T2^;Prox1^F/F^*** **mice.** (A) Radial type I adult NSCs are Nestin^+^ Gfap^+^ Blbp^+^. The number of triple Nestin^+^ Gfap^+^ Blbp^+^ cells observed in a 4-mo-old control (C) is reduced in *Nestin-CreER^T2^;Prox1^F/F^* littermates treated with TM from P0 to P15 (B, D). These results are similar to the ones shown in Figure 8A when counting radial-glia-like cells using only Nestin^+^. Data represent the mean number of positive cells per DG section ± SD. (*N* = 3 mice). Paired *t* test. *** *p*<0.001.
(3.55 MB TIF)

Figure S10 ***Hes5*** **expression is downregulated in the** ***Nestin-CreER^T2^*** **;** ***Prox1^F/F^*** **SGZ.** (A) *Hes5* expression was observed in the SGZ of 16-wk-old control mice. (B) *Hes5* expression was barely detected in the SGZ of 16-wk-old *Nestin-CreER^T2^*;*Prox1^F/F^* mice.
(2.70 MB DOC)

Figure S11 **Prox1 ectopic expression in several brain regions of** ***Nestin-Cre;Jojo-Prox1*** **embryos.** Prox1 is ectopically expressed in the ventricular, subventricular, and mantle zones of the brain of *Nestin-Cre;Jojo-Prox1* embryos like the olfactory bulb (A), lateral ventricle (F), and cortex (K). Prox1 ectopic expression in these regions does not induce premature differentiation as shown by Nestin (B, B′, G, G′, L, L′), Sox9 (C, C′, H, H′, M, M′), and β-TubIII (E, E′, J, J′, O, O′) IHC. As shown by Ki67 staining, no changes in proliferation were observed (D, D′, I, I′, N, N′). OV, Olfactory ventricle; NE, Neuroepithelium; IIIV, Third ventricle; ST, Striatum; SP, Septum; Cortex, Cx.
(2.86 MB TIF)

Figure S12 **Prox1 mis-expression promotes premature differentiation of NSCs.** Anti-C-Prox1 immunostaining shows a smaller dentate gyrus (DG) in P8 *Nestin-CreER^T2^;JoJo-Prox1* mice (B, E). There is no difference in the number of PH3^+^ cells in the DG region of control and *Nestin-CreER^T2^;JoJo-Prox1* (F) mice at P8. However, the number of Ki67^+^ cells is reduced in the *Nestin-CreER^T2^;JoJo-Prox1* DG at this stage (G). There are no differences in the number of TUNEL^+^ cells at this stage (H). Double b-Gal (red) and Nestin (I), Dcx (J), NeuN (K), Gfap (L), and NG2 (M) (blue) IHC on the DG of P8 *Nestin-CreER^T2^;JoJo-Prox1* pups shows that Prox1-misexpression induces neuronal differentiation. (N) Prox1 is ectopically expressed in a Type I Nestin^+^ cell (arrow) of adult *Nestin-Cre;JoJo-Prox1* mice. As a consequence of Prox1 ectopic expression, the number of Sox2^+^ (P) and Calretinin^+^ (R) cells is reduced in the SGZ of adult *Nestin-Cre;JoJo-Prox1* mice. Data represent the mean number of positive cells per DG section ± SD. *N* = 3 brains. Paired *t* test. ** *p*<0.01; *** *p*<0.001. Blue bars are controls. Red bars are *Nestin-Cre;JoJo-Prox1*. Scale bar: 100 µm.
(3.11 MB TIF)

## Abbreviations

DG: dentate gyrus
DNE: dentate neuroepithelium
MS: migratory stream
SGZ: subgranular zone
TM: tamoxifen
