# Supplementary figures and images for: Prox1 Is Required for Granule Cell Maturation and Intermediate Progenitor Maintenance During Brain Neurogenesis
Source: PLoS Biol. 2010 Aug 17;8(8):e1000460. doi: 10.1371/journal.pbio.1000460 (PMC2923090; doi:10.1371/journal.pbio.1000460)

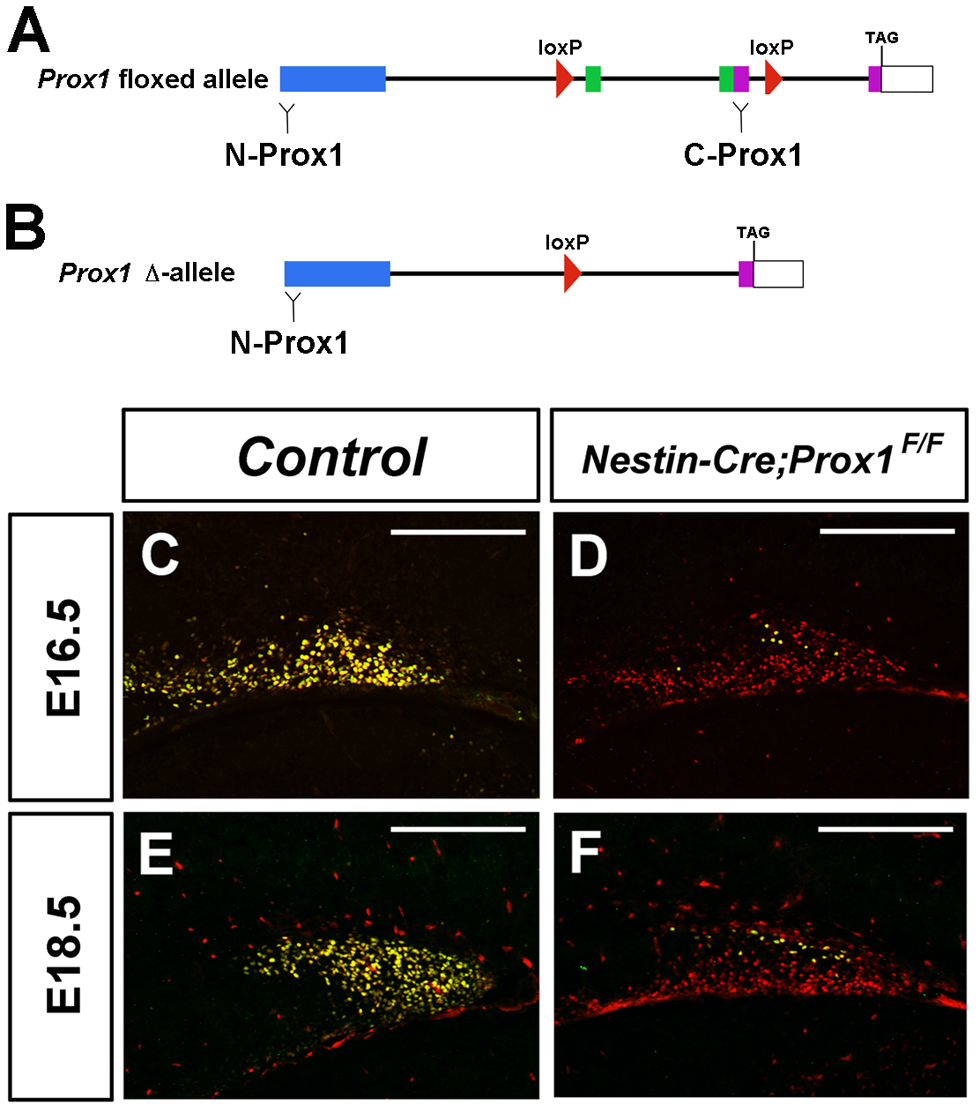

Supplement: Figure S1 — Detection of Δ-Prox1 cells in Prox1 conditional mutant mice. (A, B) Schematic representation of the different Prox1 alleles described in this article and the recognition domains of the anti-C-Prox1 and anti-N-Prox1 antibodies. (A) In the Prox1 floxed allele, loxP sites flank part of the prospero domain (purple) and the homeodomain (green). (B) Following Cre-mediated excision (Δ form), only a nonfunctional N-terminal part of Prox1 remains, without most of the prospero domain and without the entire homeodomain. Anti-N-Prox1 antibodies recognize both the wild-type and Δ forms of the Prox1 protein, but anti-C-Prox1 antibodies recognize only the wild-type full-length protein. For more details, see Hervey et al. (2005). (C–F) Coronal sections of control (C, E) and Nestin-Cre;Prox1F/F (D, F) dentate gyrus at different developmental stages show the presence of the Δ-Prox1+ cell population in the conditional-mutant mice. Sections were immunolabeled with antibodies against N-Prox1+ (red) and C-Prox1+ (green). (0.64 MB TIF) [file pbio.1000460.s001.tif]

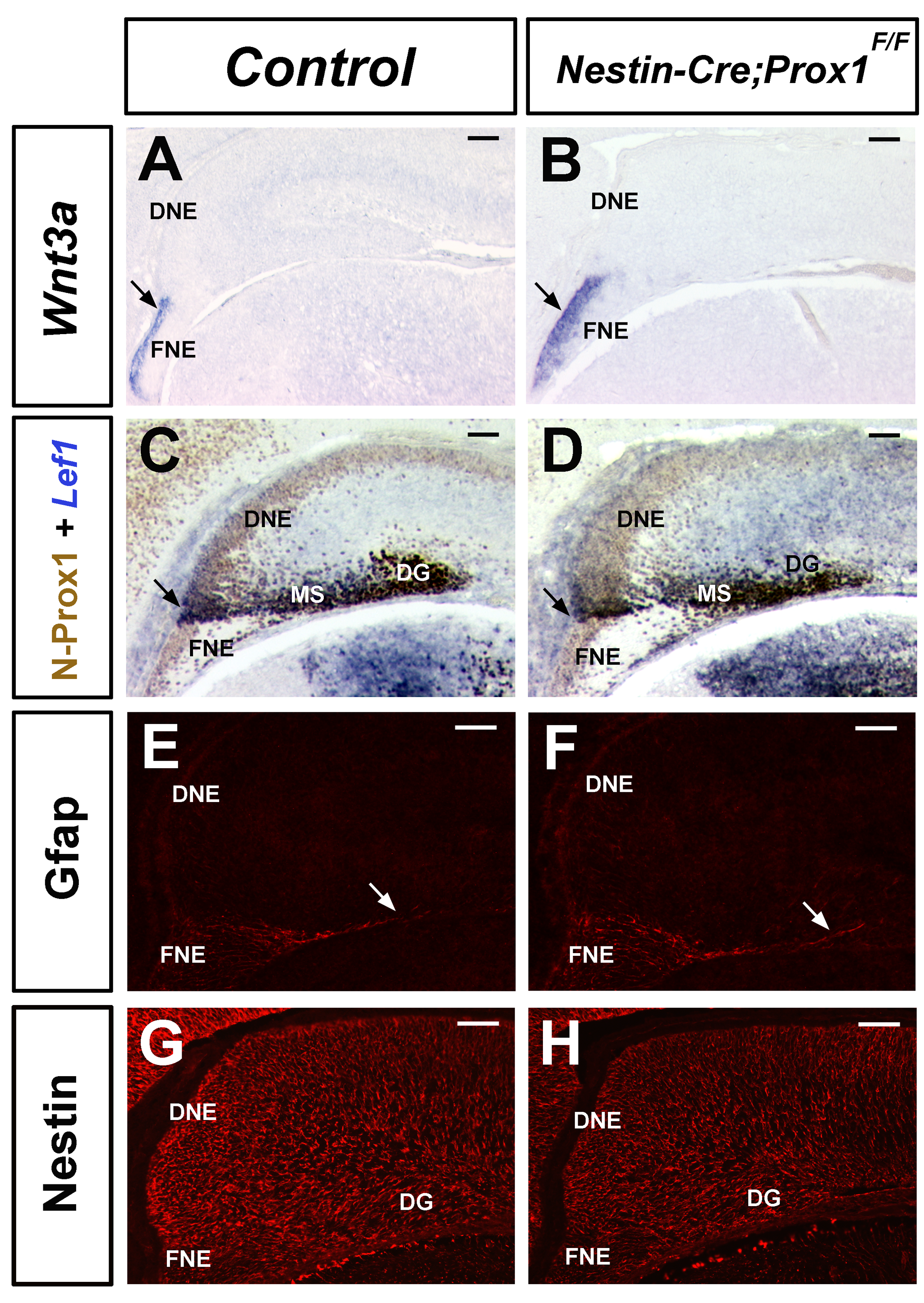

Supplement: Figure S2 — N-Prox1+ cells migrate from the dentate neuroepithelium to the region of the dentate gyrus in Nestin-Cre;Prox1F/F mice. (A, B) Wnt3a is expressed in the fimbria neuroepithelium (FNE) (arrows) of E16.5 control and Nestin-Cre;Prox1F/F brains. (C, D) In response to Wnt3a signaling from the FNE, Lef1 is expressed in N-Prox1+ migrating cells (arrows) in E16.5 control and mutant embryos. The radial glia scaffolding is normal in the Nestin-Cre;Prox1F/F dentate gyrus (DG), as shown by Gfap (E, F) and Nestin (G, H) immunostaining. DNE, Dentate neuroepithelium; MS, Migratory stream. Scale bar in (M–T): 50 µm. (6.42 MB TIF) [file pbio.1000460.s002.tif]

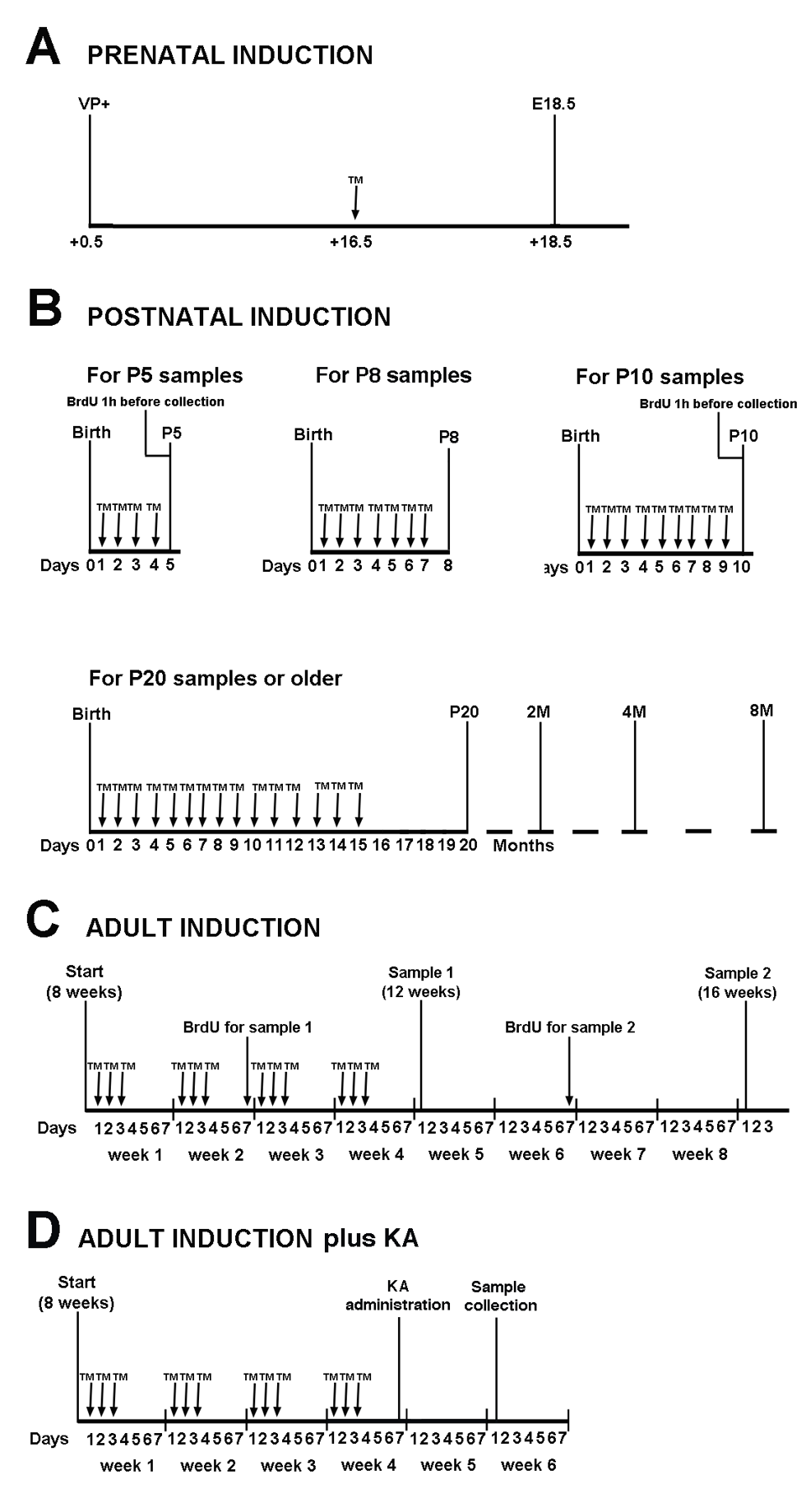

Supplement: Figure S3 — Schematic representation of the TM administration protocol. (A) For prenatal induction, TM was administered to time-mated female mice at E16.5 and embryos were harvested at E18.5. (B) For postnatal induction, pups were fed daily with TM until collection day or P15. (C) For adult induction, 8-wk-old mice were treated with TM for 4 wk, 3 d a week. Samples were collected 4 wk or 8 wk after the beginning of TM treatment. (D) At the end of the TM treatment, kainic acid was administered to control and conditional mutant mice. The samples were collected 8 d after KA administration. (0.28 MB TIF) [file pbio.1000460.s003.tif]

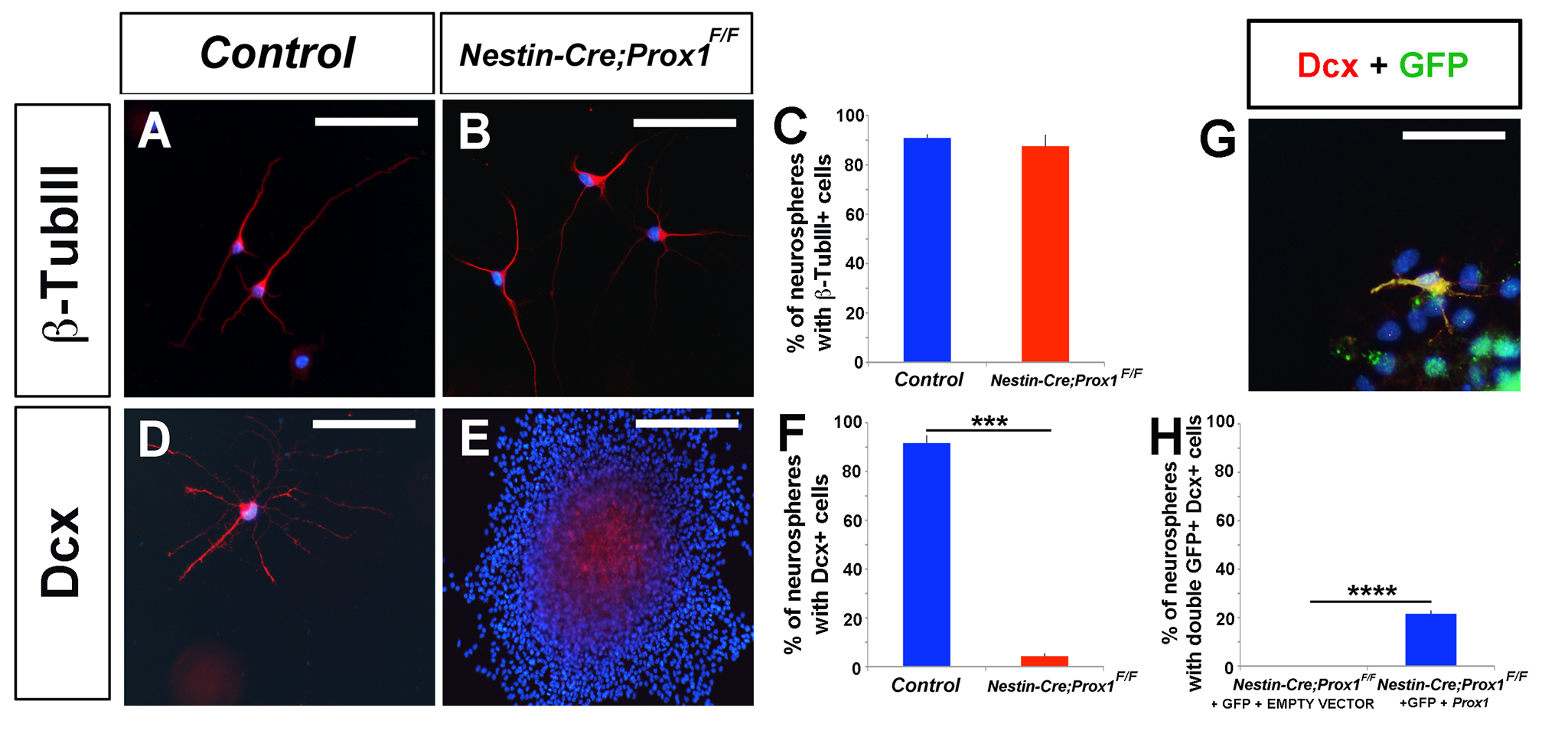

Supplement: Figure S4 — Prox1 is necessary for neuronal differentiation in vitro. (A–C) Neurospheres generated from the hippocampus of E16.5 control and Nestin-Cre;Prox1F/F mice are capable of producing βTub-III+ neurons (N = 3 experiments; 175 control and 135 Nestin-Cre;Prox1F/F neurospheres were analyzed). (D–F) However, most of the neurospheres obtained from Nestin-Cre;Prox1F/F mice failed to generate Dcx+ cells (N = 3 experiments; 216 control and 190 Nestin-Cre;Prox1F/F neurospheres were analyzed). (G, H) 3 d after co-transfection with GFP- and Prox1-expressing plasmids, around 20% of the Nestin-Cre;Prox1F/F GFP+ neurospheres generated Dcx+ neurons (N = 3 experiments; a total of 76 Nestin-Cre;Prox1F/F GFP+ neurospheres and 73 GFP+ Nestin-Cre;Prox1F/F neurospheres transfected with Prox1 were analyzed). Data represent the mean number of the percentage of neurospheres per experiment ± SD. Paired t test. *** p<0.001; **** p<0.0001. Scale bar in (A, B, D): 50 µm. Scale bar in (E): 100 µm. Scale bar in (G): 25 µm. (1.52 MB TIF) [file pbio.1000460.s004.tif]

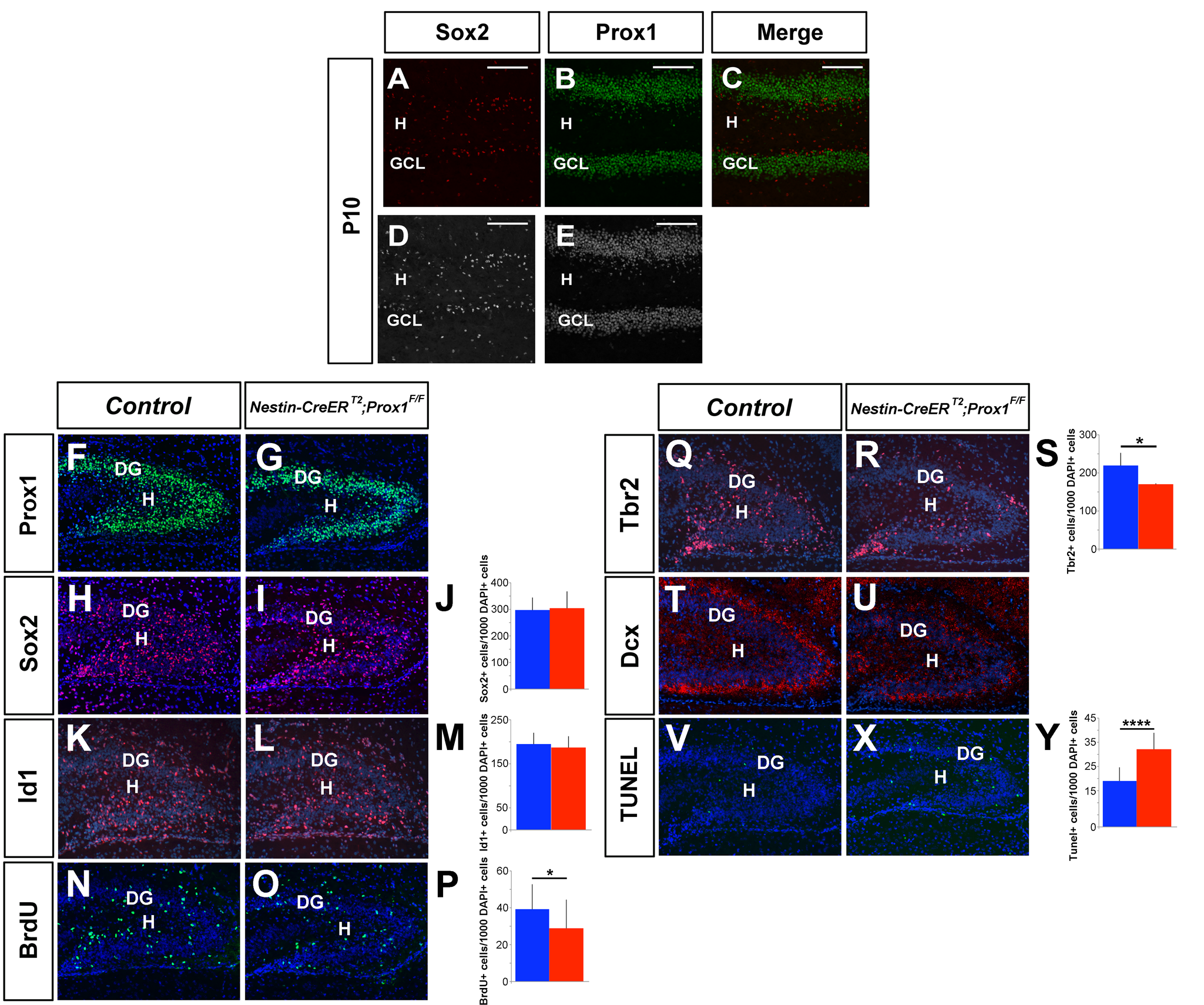

Supplement: Figure S5 — The number of intermediate progenitors and maturing granule cells is reduced in the dentate gyrus of P5 Nestin-Cre;Prox1F/F pups treated with TM from P0 to P5. (A–E) Sox2+ Prox1+ cells are not detected in the wild-type dentate gyrus (DG) at P10. (F) C-Prox1+ cells are present in the control DG and the hilus (H) at P5. (G) TM administration from P0 to P5 reduces the number of C-Prox1+ cells in the hilus of P5 Nestin-CreERT2;Prox1F/F pups. However, no alterations in the number of Sox2+ (H–J) or Id1+ PECAM− (K–M) NSCs were detected in Nestin-CreERT2;Prox1F/F mutant brains. (N–P) Following a 1-h pulse, BrdU immunostaining revealed a small reduction in proliferation in the DG of Nestin-CreERT2;Prox1F/F brains. (Q–S) As shown by Tbr2 staining, the number of intermediate progenitors is reduced in the DG of the conditional mutant mice at this stage. (T, U) The number of Dcx+ cells is also reduced in the DG of Nestin-CreERT2;Prox1F/F pups at P5. An increase in the number of TUNEL+ cells (V–Y) is observed in the DG of Nestin-CreERT2;Prox1F/F brains at P5. Data represent the mean number of positive cells per DG section ± SD (N = 3 mice). Paired t test. * p<0.1; **** p<0.0001. GCL, granule cell layer; SGZ, Subgranular zone. Scale bar: 100 µm. (2.97 MB TIF) [file pbio.1000460.s005.tif]

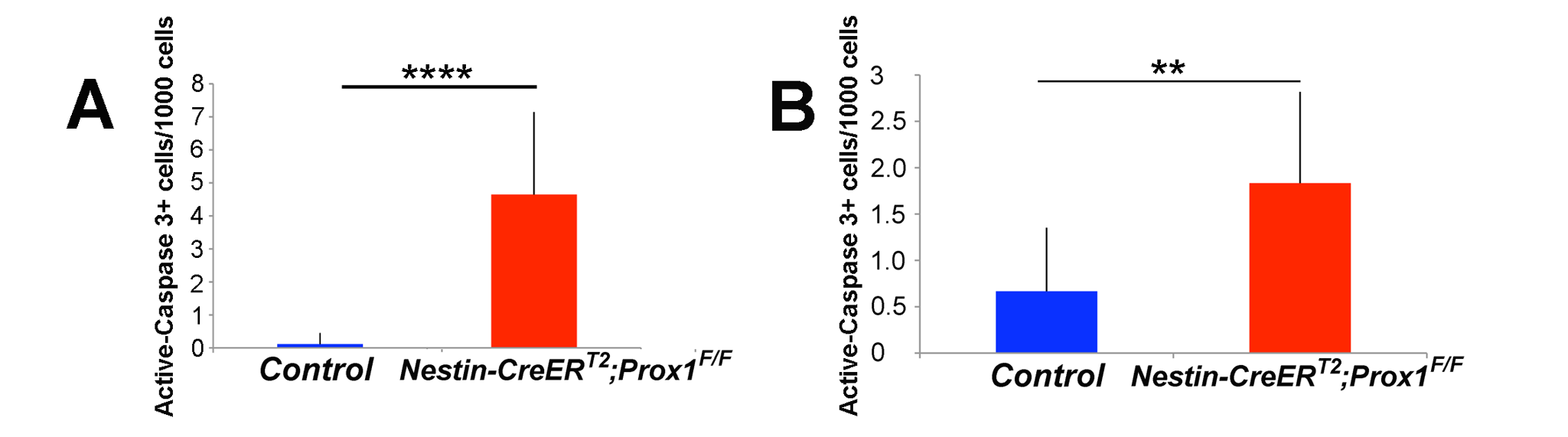

Supplement: Figure S6 — The dentate gyrus of TM-treated Nestin-CreERT2;Prox1F/F pups exhibits an increased number of active caspase-3+ cells at P5 and P10. The number of active caspase-3+ cells is increased in the dentate gyrus of P5 (A) and P10 (B) TM-treated Nestin-CreERT2;Prox1F/F pups. Data represent the mean number of positive cells per DG section ± SD (N = 3 mice). Paired t test. ** p<0.01; **** p<0.0001. (0.12 MB TIF) [file pbio.1000460.s006.tif]

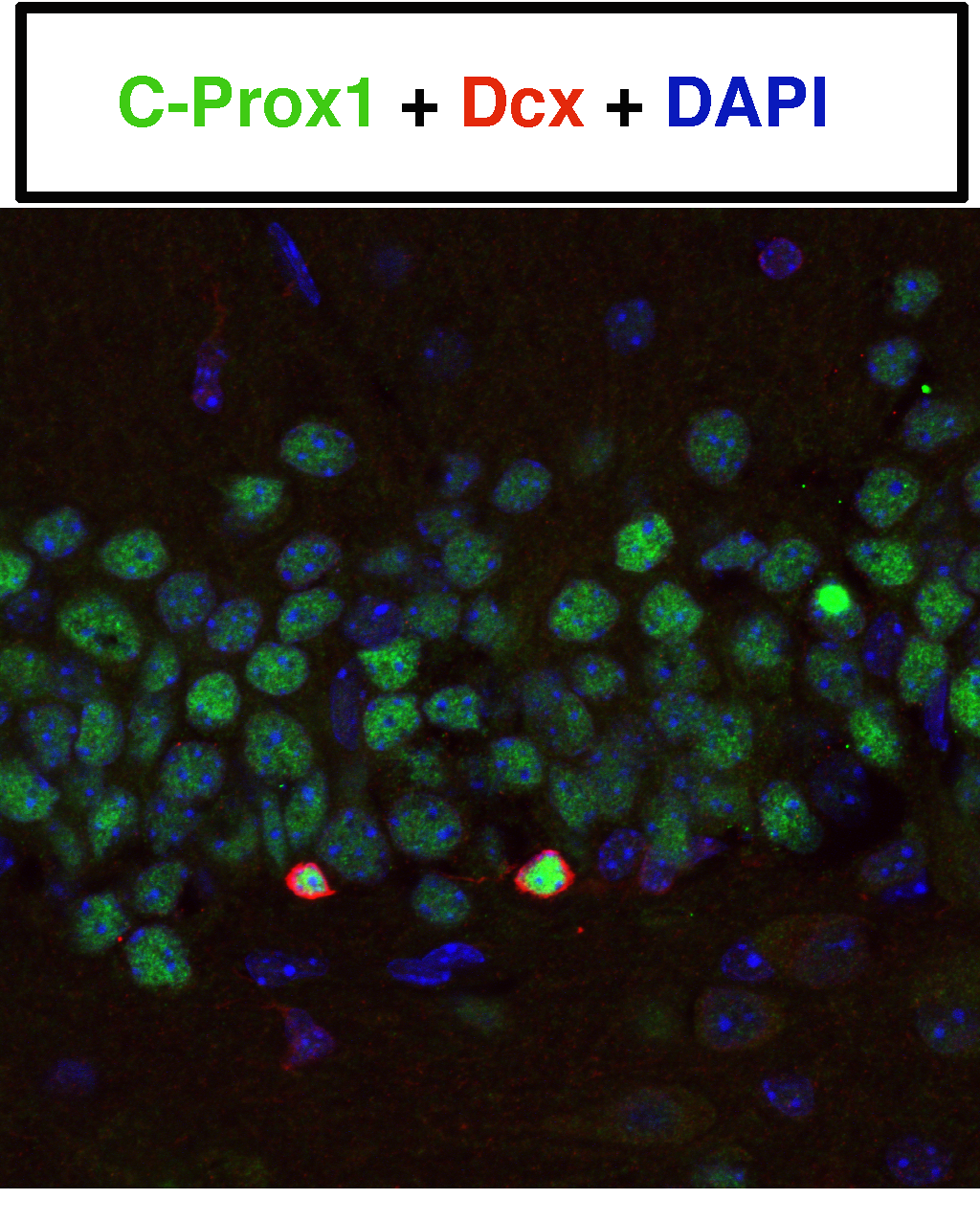

Supplement: Figure S7 — Dcx+ cells remaining in TM-treated Nestin-CreERT2;Prox1F/F mice are those that have escaped Cre-mediated deletion. Double C-Prox1/Dcx immunostaining shows that in 2-mo-old Nestin-CreERT2;Prox1F/F mice treated with TM from P0 to P15 the remaining Dcx+ cells were also C-Prox1+. (1.97 MB TIF) [file pbio.1000460.s007.tif]

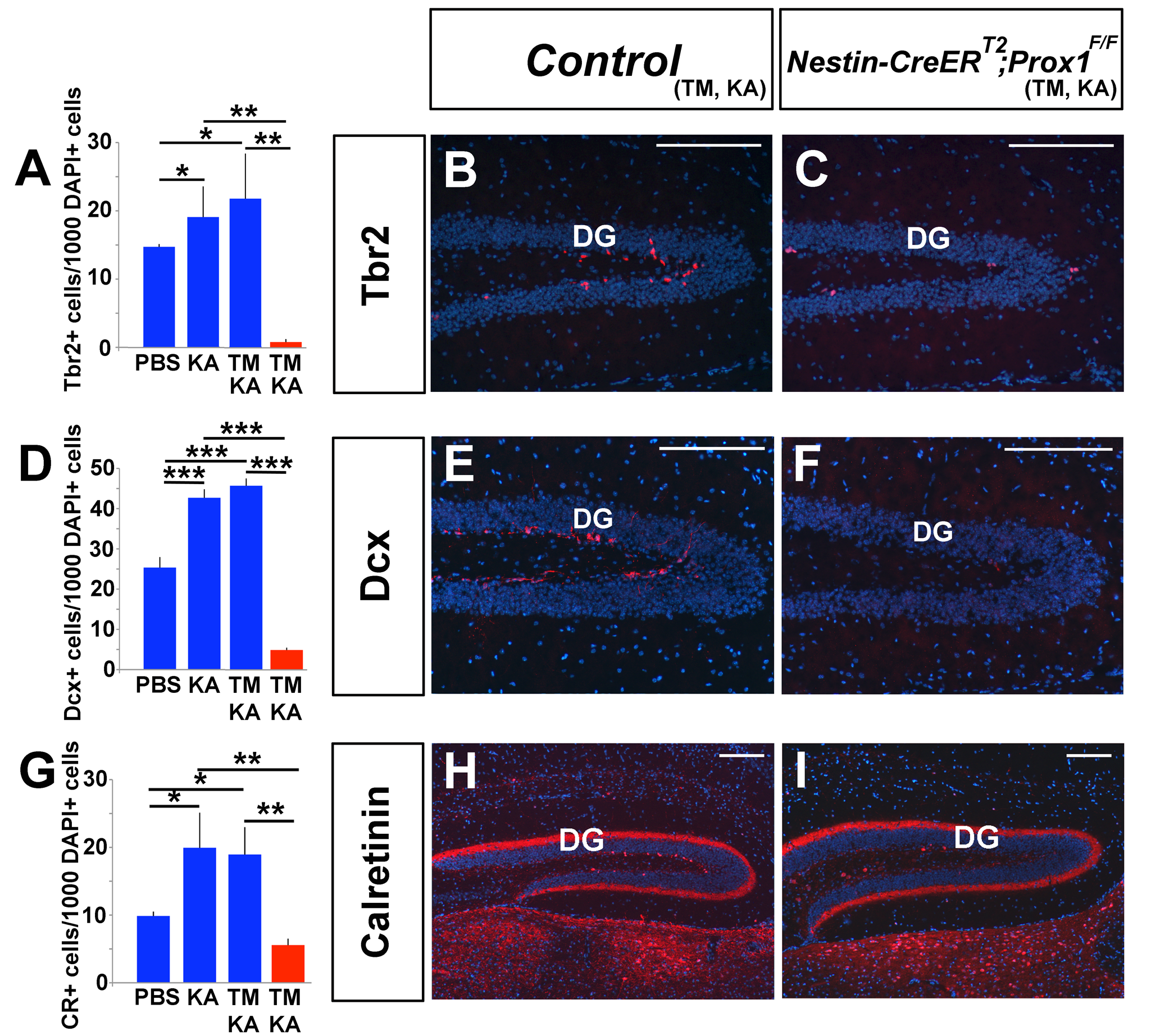

Supplement: Figure S8 — Kainic Acid do not induce adult neurogenesis in the SGZ of TM-treated Nestin-CreERT2;Prox1F/F mice. (A, B) The number of Tbr2 intermediate progenitors is increased in the dentate gyrus (DG) of control mice treated with TM or with TM and Kainic Acid (KA). However, KA is not able to increase the number of Tbr2+ cells in 12-wk-old TM-treated Nestin-CreERT2;Prox1F/F mice (A, C). Similar results were observed with Dcx+ (D–F) and Calretinin+ (G–I) cells. Data represent the mean number of positive cells per DG section ± SD (N = 3 mice). Paired t test. * p<0.1; ** p<0.01; *** p<0.001. Scale bar: 100 µm. (3.77 MB TIF) [file pbio.1000460.s008.tif]

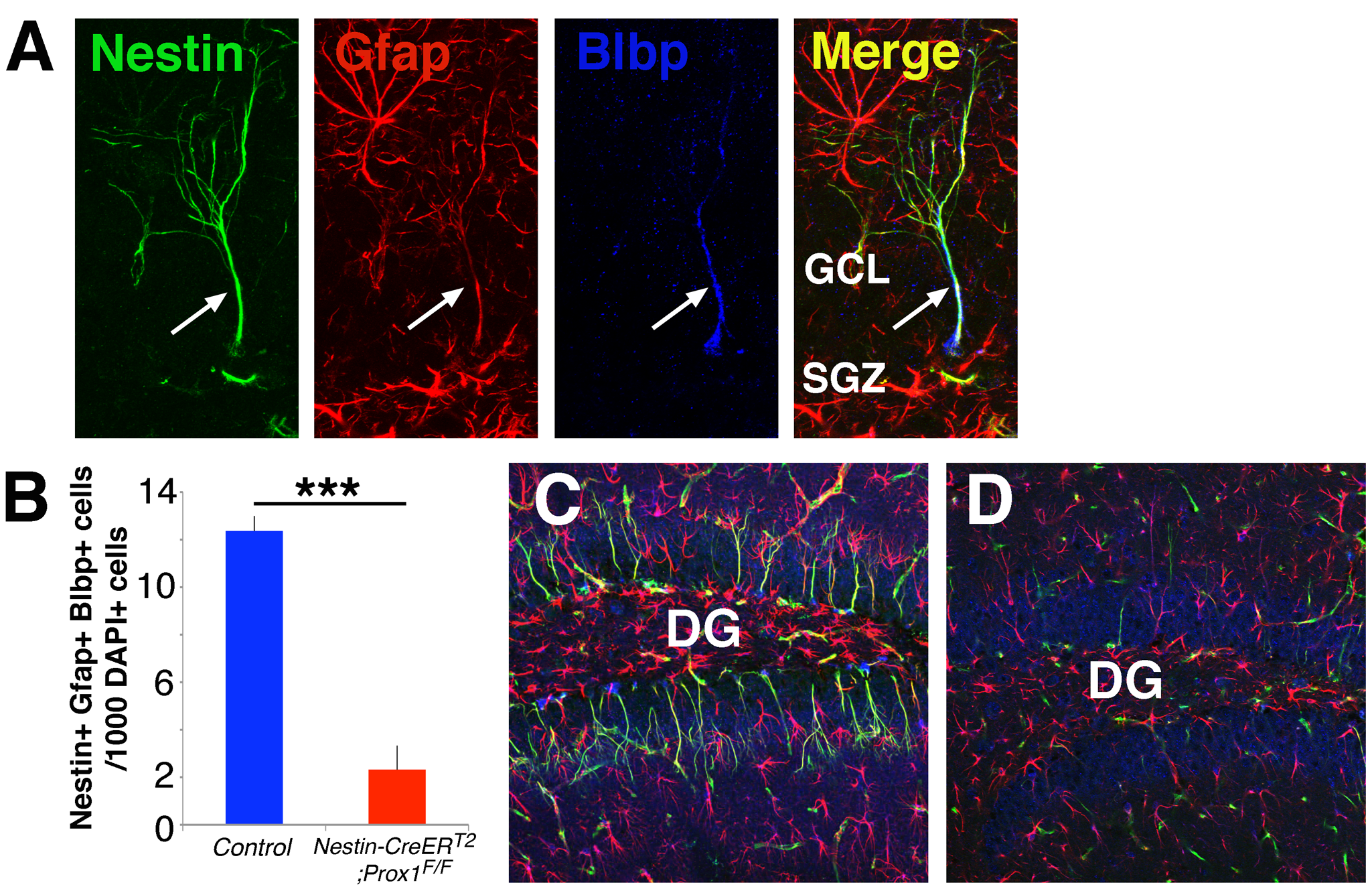

Supplement: Figure S9 — Analysis of the NSC population in TM-treated Nestin-CreERT2;Prox1F/F mice. (A) Radial type I adult NSCs are Nestin+ Gfap+ Blbp+. The number of triple Nestin+ Gfap+ Blbp+ cells observed in a 4-mo-old control (C) is reduced in Nestin-CreERT2;Prox1F/F littermates treated with TM from P0 to P15 (B, D). These results are similar to the ones shown in Figure 8A when counting radial-glia-like cells using only Nestin+. Data represent the mean number of positive cells per DG section ± SD. (N = 3 mice). Paired t test. *** p<0.001. (3.55 MB TIF) [file pbio.1000460.s009.tif]

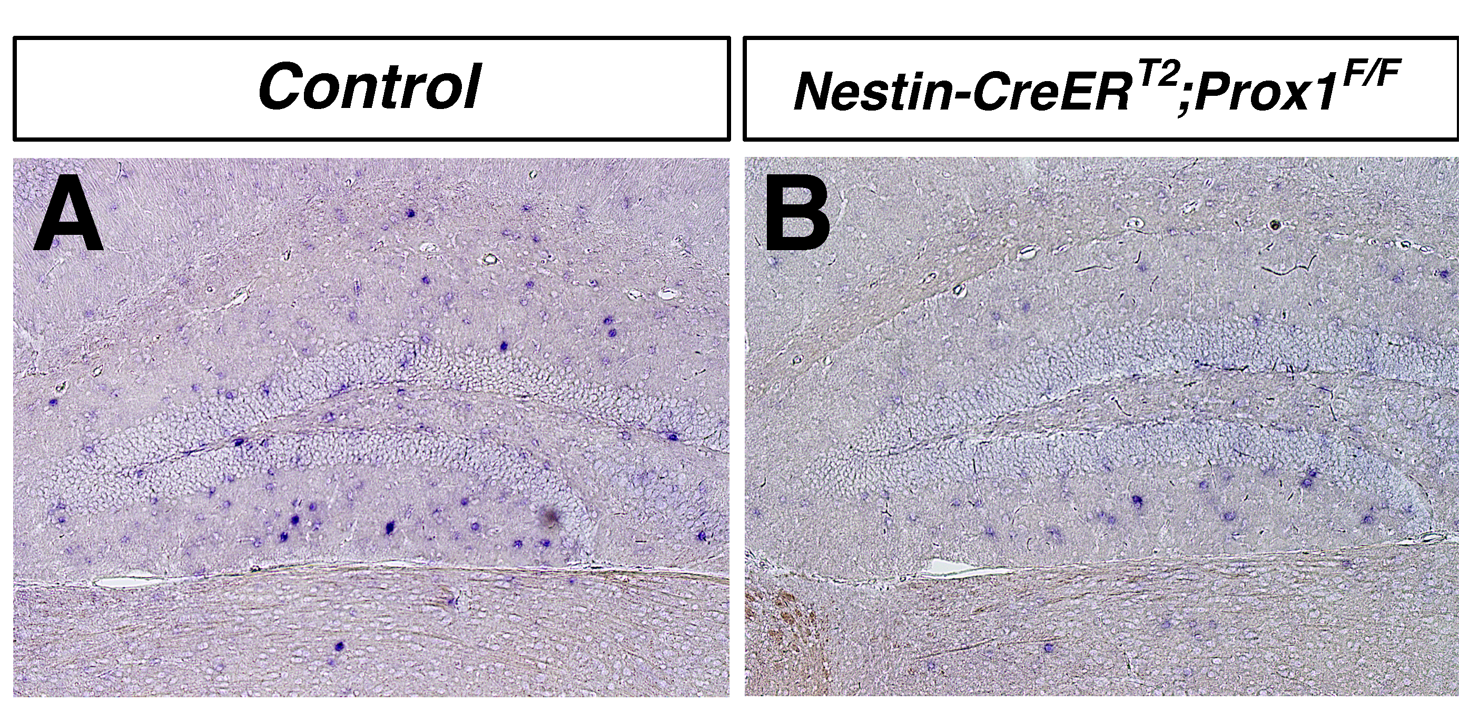

Supplement: Figure S10 — Hes5 expression is downregulated in the Nestin-CreERT2 ; Prox1F/F SGZ. (A) Hes5 expression was observed in the SGZ of 16-wk-old control mice. (B) Hes5 expression was barely detected in the SGZ of 16-wk-old Nestin-CreERT2;Prox1F/F mice. (2.70 MB DOC) [file pbio.1000460.s010.tif]

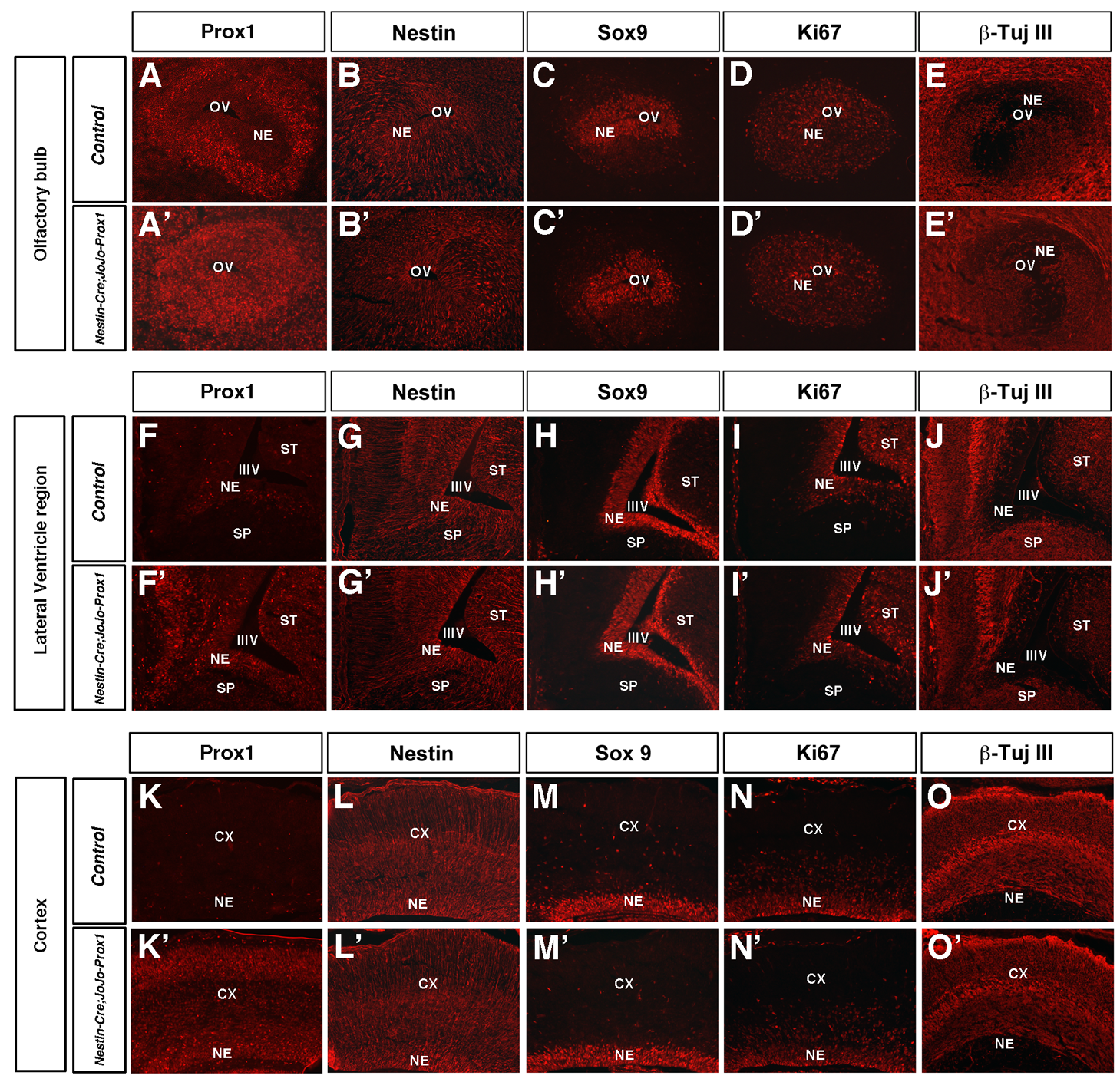

Supplement: Figure S11 — Prox1 ectopic expression in several brain regions of Nestin-Cre;Jojo-Prox1 embryos. Prox1 is ectopically expressed in the ventricular, subventricular, and mantle zones of the brain of Nestin-Cre;Jojo-Prox1 embryos like the olfactory bulb (A), lateral ventricle (F), and cortex (K). Prox1 ectopic expression in these regions does not induce premature differentiation as shown by Nestin (B, B′, G, G′, L, L′), Sox9 (C, C′, H, H′, M, M′), and β-TubIII (E, E′, J, J′, O, O′) IHC. As shown by Ki67 staining, no changes in proliferation were observed (D, D′, I, I′, N, N′). OV, Olfactory ventricle; NE, Neuroepithelium; IIIV, Third ventricle; ST, Striatum; SP, Septum; Cortex, Cx. (2.86 MB TIF) [file pbio.1000460.s011.tif]

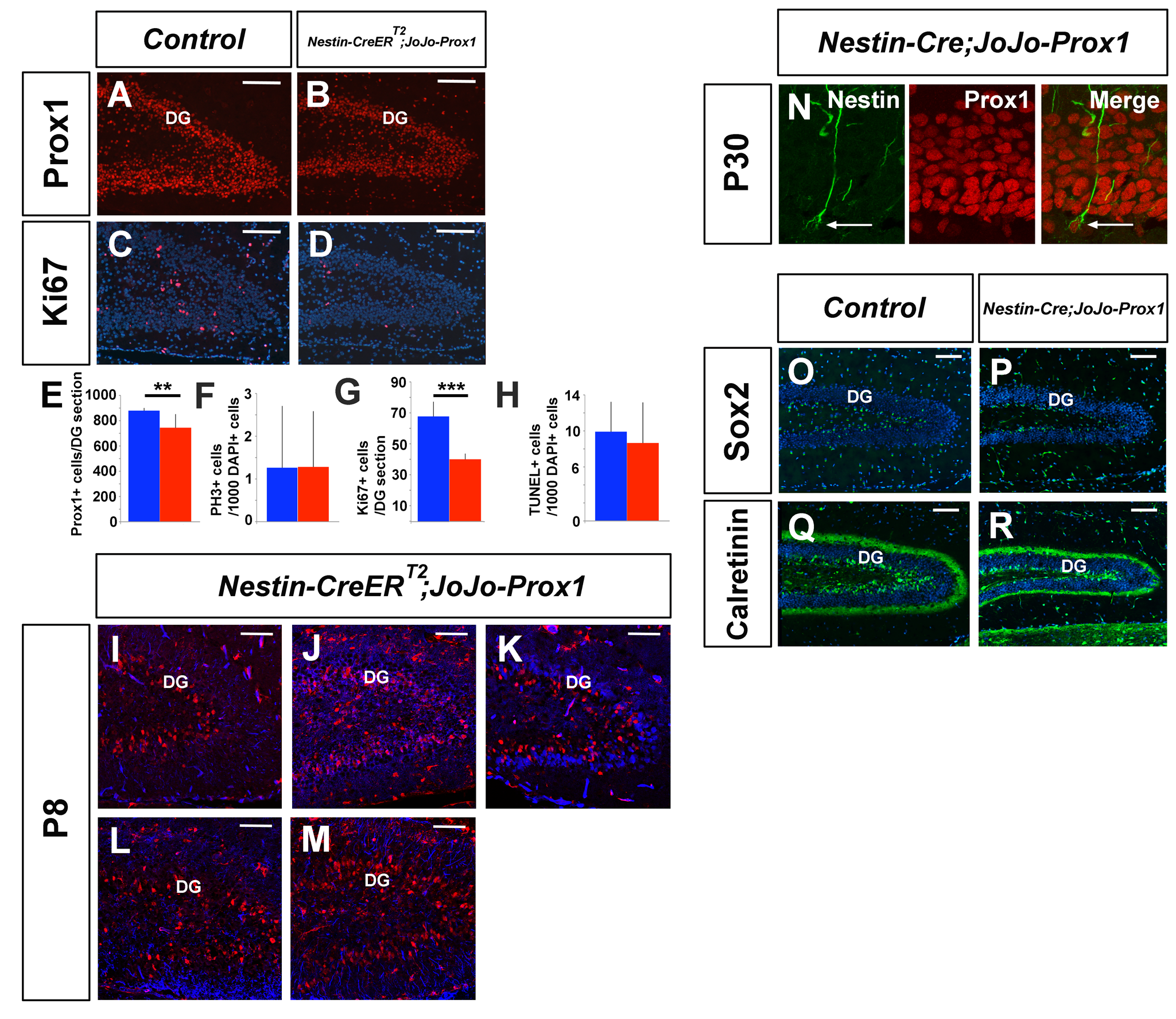

Supplement: Figure S12 — Prox1 mis-expression promotes premature differentiation of NSCs. Anti-C-Prox1 immunostaining shows a smaller dentate gyrus (DG) in P8 Nestin-CreERT2;JoJo-Prox1 mice (B, E). There is no difference in the number of PH3+ cells in the DG region of control and Nestin-CreERT2;JoJo-Prox1 (F) mice at P8. However, the number of Ki67+ cells is reduced in the Nestin-CreERT2;JoJo-Prox1 DG at this stage (G). There are no differences in the number of TUNEL+ cells at this stage (H). Double b-Gal (red) and Nestin (I), Dcx (J), NeuN (K), Gfap (L), and NG2 (M) (blue) IHC on the DG of P8 Nestin-CreERT2;JoJo-Prox1 pups shows that Prox1-misexpression induces neuronal differentiation. (N) Prox1 is ectopically expressed in a Type I Nestin+ cell (arrow) of adult Nestin-Cre;JoJo-Prox1 mice. As a consequence of Prox1 ectopic expression, the number of Sox2+ (P) and Calretinin+ (R) cells is reduced in the SGZ of adult Nestin-Cre;JoJo-Prox1 mice. Data represent the mean number of positive cells per DG section ± SD. N = 3 brains. Paired t test. ** p<0.01; *** p<0.001. Blue bars are controls. Red bars are Nestin-Cre;JoJo-Prox1. Scale bar: 100 µm. (3.11 MB TIF) [file pbio.1000460.s012.tif]
